# Involvement of SUT1 and SUT2 Sugar Transporters in the Impairment of Sugar Transport and Changes in Phloem Exudate Contents in Phytoplasma-Infected Plants

**DOI:** 10.3390/ijms22020745

**Published:** 2021-01-13

**Authors:** Federica De Marco, Brigitte Batailler, Michael R. Thorpe, Frédérique Razan, Rozenn Le Hir, Françoise Vilaine, Alain Bouchereau, Marie-Laure Martin-Magniette, Sandrine Eveillard, Sylvie Dinant

**Affiliations:** 1 Institut Jean-Pierre Bourgin, INRAE, AgroParisTech, Université Paris-Saclay, 78000 Versailles, France; demarco.federica@gmail.com (F.D.M.); rozenn.le-hir@inrae.fr (R.L.H.); francoise.vilaine@inrae.fr (F.V.); 2 UMR 1332 Biologie du Fruit et Pathologie, INRAE et Université de Bordeaux, CS20032, 33140 Villenave d’Ornon CEDEX, France; brigitte.batailler@laposte.net (B.B.); frederique971@live.fr (F.R.); sandrine.eveillard@inrae.fr (S.E.); 3 Plant Science Division, Research School of Biology, The Australian National University, Canberra, ACT 0200, Australia; michael.thorpe@anu.edu.au; 4 UMR IGEPP, INRAE, Agrocampus Ouest, Université de Rennes 1, 35653 Le Rheu CEDEX, France; alain.bouchereau@univ-rennes1.fr; 5 Institute of Plant Sciences Paris-Saclay (IPS2), Université Paris-Saclay, CNRS, INRAE, Univ Evry, 91405 Orsay, France; marie_laure.martin-magniette@agroparistech.fr; 6 Institute of Plant Sciences Paris-Saclay (IPS2), Université de Paris, CNRS, INRAE, 91405 Orsay, France; 7 UMR MIA-Paris, AgroParisTech, INRAE, Université Paris-Saclay, 75005 Paris, France

**Keywords:** phloem, peroxisome, sugar metabolism, glyoxylate, glycolate, source-sink relationships, carbon allocation, photorespiration, metabolome, plant-pathogen interaction, phytoplasma, defense

## Abstract

Phytoplasmas inhabit phloem sieve elements and cause abnormal growth and altered sugar partitioning. However, how they interact with phloem functions is not clearly known. The phloem responses were investigated in tomatoes infected by “*Candidatus* Phytoplasma solani” at the beginning of the symptomatic stage, the first symptoms appearing in the newly emerged leaf at the stem apex. Antisense lines impaired in the phloem sucrose transporters SUT1 and SUT2 were included. In symptomatic sink leaves, leaf curling was associated with higher starch accumulation and the expression of defense genes. The analysis of leaf midribs of symptomatic leaves indicated that transcript levels for genes acting in the glycolysis and peroxisome metabolism differed from these in noninfected plants. The phytoplasma also multiplied in the three lower source leaves, even if it was not associated with the symptoms. In these leaves, the rate of phloem sucrose exudation was lower for infected plants. Metabolite profiling of phloem sap-enriched exudates revealed that glycolate and aspartate levels were affected by the infection. Their levels were also affected in the noninfected *SUT1*- and *SUT2*-antisense lines. The findings suggest the role of sugar transporters in the responses to infection and describe the consequences of impaired sugar transport on the primary metabolism.

## 1. Introduction

In their host plants, phytoplasmas represent an interesting case of obligate bacterial pathogens inhabiting the sieve elements (SE) and transmitted by phloem-feeding insects. Phytoplasmas cause diseases affecting crops worldwide, provoking huge economic losses [1,2]. Since they multiply exclusively in the SEs, phytoplasma propagate systemically from the site of infection to sink organs, a process largely explained by convection, along with the assimilate flow in the phloem [3,4], even if the movement of phytoplasmas cannot be solely explained by mass flow [5,6]. The infection dynamic depends on the plant–phytoplasma pathosystem [3,4,7,8,9]. The symptomatology of the disease is not solely determined by phytoplasma titer; it varies with environmental factors and plant age at the time of infection [10], suggesting complex interplays with the host physiology. Phytoplasmas lack many genes of the core metabolic processes, leading to auxotrophy for many nutrients that must be supplied by the highly specialized phloem environment [11].

Among the consequences of the infection that are observed in infected plants, the most frequent are the disruption of photoassimilate distributions [12,13], increased or decreased sugars and starch in source or sink leaves, depending on the pathosystem [14,15,16,17,18]; altered accumulation of amino acids, organic acids and secondary metabolites [18,19,20,21]; impairment of the photosynthetic processes [15,17,18,22,23,24] and production of H_2_O_2_ and activation of the antioxidant defense system [25,26,27].

Callose deposition at sieve plates and aggregations of SE protein filaments, leading potentially to SE occlusions [28,29,30], are also frequently observed in plants infected by phytoplasma. Studies on symptomatic, well-established phytoplasma infections reported disorganization of the vascular tissues [4,31] and the transcriptional reprogramming of genes involved in sugar transport and metabolism [16,18,24,28,32,33]. A phytoplasma infection also triggers modifications in the phloem sap composition [25,34,35,36], with changes in metabolites produced by diverse metabolic pathways. Such effects could be triggered by the phytoplasma for nutrition, plant defense response or physiological adjustments of impaired phloem activity [5]. Finally, phytoplasmas secrete effectors that spread laterally from the SE [37,38,39]. The early steps of the infection, however, are poorly known. It would be helpful to better understand how these bacteria affect their host’s metabolism and phloem function.

The Stolbur phytoplasma—tomato pathosystem is a common model for studying plant–phytoplasma interactions. “*Candidatus* Phytoplasma solani”, the phytoplasma responsible for Stolbur disease, belongs to the 16SrXII group [40]. Several strains infect tomatoes, the phytoplasma (PO) strain causing severe symptoms, including leaf stunting and abnormal floral buds and flowers, associated with an activation of salicylic acid-mediated defense responses, such as *PR1a* and *PR2a* [41]. Phloem hyperplasia and callose deposits are present in symptomatic leaves [42], and phytoplasma accumulates massively into infected SEs [43,44]. As for other plant–phytoplasma interactions, the infection affects sugar homeostasis, with alterations of sucrose synthase and invertase activities in both mature and young leaves of Stolbur-infected plants [45].

Sugar metabolism and phloem transport are well-documented for tomatoes. Sucrose loading into the phloem involves transporter-mediated sucrose transfer from the apoplasm into the SEs, so-called apoplasmic loading. Sucrose is loaded in the minor veins by Sucrose Transporter 1 (SUT1), a high-affinity sucrose proton symporter localized to the plasma membrane of the SEs [46]. The low-affinity sucrose transporter SUT2 is necessary for unloading in some sink organs, such as fruits, at some stages during their development [47]. In the midribs and on the entire length of the axial pathway from source to sink, i.e., along the transport phloem, SUT1 or SUT2 could regulate sugar release and retrieval [48] and potentially contribute to the release/retrieval equilibrium, depending on the stages of development [49]. Phloem loading fluctuates depending on the environment, with SUT1 likely involved in these regulations [50]. Other classes of sugar transporters, such as sugar facilitators from the Sugar Will Eventually Be Exported Transporters family (SWEET), act on intercellular and intracellular sugar translocation, some acting in cells near the sieve elements [51], and many more transporters enable exchanges of sugars or other metabolites between sieve elements and the surrounding cells along the phloem pathway [52]. Companion cell metabolism can also affect sieve element metabolite contents because of their connection via plasmodesmata. Several key enzymes have been characterized in vascular cells, such as fructokinases (FRK), which regulate the pools of fructose and sucrose, and participate in the physiology and development of the vascular tissues [53,54]. The regulation of sugar metabolism is expected to be highly coordinated with sugar transport. However, information regarding the impact of a disruption of phloem transport on the translocation of other classes of primary metabolites, such as amino acids and organic acids, is still limited. It is also unclear whether phytoplasma infection interferes with sugar transport and companion cell metabolism. Defects in carbon allocation observed in infected plants could result either from an impairment of sugar transport due to reduced sucrose loading or altered release in the surrounding tissues or from occlusion of the sieve tubes by callose deposits.

In this study, we investigated the role of *SUT1* and *SUT2* in the disruption of photoassimilate distribution observed in phytoplasma-infected plants. We analyzed the early responses of tomato plants infected by “*Candidatus* Phytoplasma solani” to get clues on the events leading to Stolbur infection on alterations of phloem transport and sugar homeostasis. To determine whether the infection alters sugar phloem transport, regulated by the sucrose transporters SUT1 and SUT2, we included in our study the two antisense lines silenced for *SUT1* and *SUT2* [55]. In order to get a more comprehensive view of the metabolic pathways that are altered during the infection in the phloem, we analyzed the metabolite profile of the phloem sap-enriched exudates, and we studied separately the effects of infection on the overall phloem flow. Altogether, our results showed that early plant responses to phytoplasma infection are associated with a *SUT1*-dependent perturbation of the translocation of photoassimilates in the phloem and with the impairment of glycolate–glyoxylate metabolism.

## 2. Results

### 2.1. Symptoms and Presence of Bacteria of the Wild-Type (WT) and of SUT1 and SUT2 Antisense Lines to the Infection

**Figure 1:**
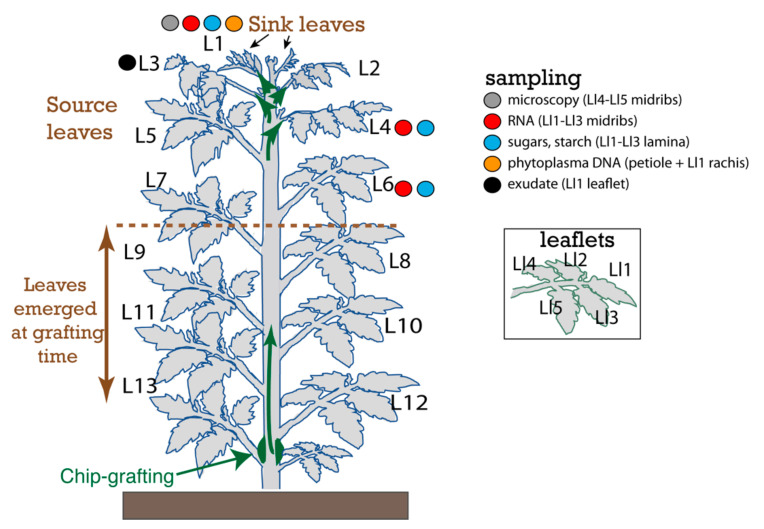
Experimental Design. Schematic representation of tomato plants when material from leaves L1, L3, L4 and L6 was collected for RNA, DNA, sugar and starch analyses and sap collection by exudation and imaging. Source and sink status of the leaves follow leaf development and expansion. In this study, leaves that are more than 60% fully expanded were considered as sources, based on the study of Turgeon (1989), who established that leaves begin to export when they are 30–60% fully expanded [56]. The L1 leaf just emerged and began to unfold at the sampling stage and was considered as a sink, with leaves L3 and older as sources and L2 indeterminate. The arrow in green indicates the direction of migration of phytoplasmas from the grafted area to the apical leaves. Based on the ages of leaves in which phytoplasma were detected at 18 days after grafting (L1 to L4) and the number of leaves that emerged after grafting (6 new leaves), it is likely that it took at least one week for graft union to be successful and for the phytoplasma to enter the translocation stream. Samplings and observations are indicated as solid circles: plant and phytoplasma RNA sampling (red circle), phytoplasma DNA sampling (orange circle), sugars and starch sampling (blue circle), imaging by transmission electron microscopy or light microscopy (grey circle) and exudate, phloem sap-enriched exudate sampling for the metabolomics analysis (black circle). Inset shows leaflets numbering within a leaf (Ll1–Ll5).

For the experiments, tomato plants from wild-type (WT), *SUT1-AS* (antisense) and *SUT2-AS* lines were inoculated with Stolbur phytoplasma strain PO by side-grafting at two alternate positions on the main stem by using scions each 3 cm from infected WT plants. Control plants were grafted with healthy scions. A delay was required for the graft to be effective, permit the propagation of the phytoplasma from the grafted zone to sink organs and cause symptoms. The responses in tomatoes infected by Stolbur phytoplasma were investigated at the beginning of the symptomatic stage of infection both in symptomatic and asymptomatic leaves, the first symptoms appearing in the younger, upper L1 leaves at the plant apex (Figure 1).

**Figure 2:**
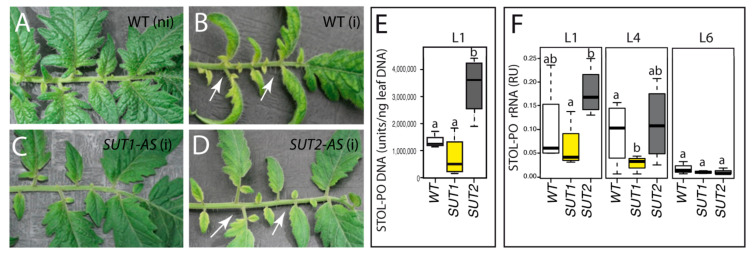
Symptoms and Stolbur phytoplasma proliferation in infected plants. (**A**–**D**) Details of L1 leaves from grafted noninfected wild-type (WT) (**A**), infected WT (**B**), infected *SUT1*-AS (antisense) (**C**) and infected *SUT2*-AS (**D**). ni: noninfected and i: infected (**D**). White arrows in (**B**,**D**) indicate leaf typical yellowing and growth reduction. (**E**,**F**) Boxplot showing phytoplasma DNA in L1 leaves in (**E**) and rRNA amounts in L1, L4 and L6 leaves in (**F**); RU: relative units of content. The box and whisker plots in (**E**,**F**) show the distribution of the biological replicates. Inside black lines represent medians; top and bottom ends of the boxes represent the first and the third quartiles, respectively; *n* = 4. Different letters denote statistically different values determined by ANOVA and Tukey’s test.

The only visible symptoms in infected WT and *SUT2*-AS plants were the beginning of yellowing, a slight crooked shape and reduced growth of the L1 leaf (Figure 2 and Table S1) and were milder than those that occur at later stages of infection [41]. In contrast, infected *SUT1*-AS plants showed mild-to-no symptoms (Figure 2C and Table S1), even though phytoplasmas were present in the L1 leaves of infected plants in all three genotypes, with higher values in *SUT2*-AS (Figure 2E,F). The L4 leaves showed no symptoms, despite the presence of phytoplasmas there (Figure 2F). The L4 leaves of *SUT1*-AS plants had low bacterial rRNA, confirming a difference in the susceptibility of this genotype. This difference persisted during the following two weeks on *SUT1*-AS plants with weaker symptoms (Table S1). In the sixth leaf (L6), which only just emerged at the time of grafting, only traces of Stolbur rRNA were found in all three genotypes.

### 2.2. Ultrastructure of the Phloem in Infected Leaves

**Figure 3:**
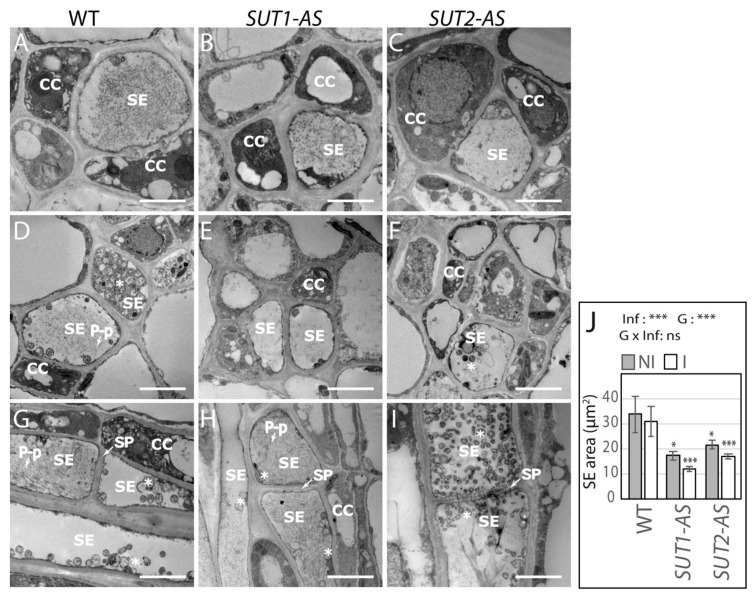
Ultrastructure of the phloem in response to the infection in the L1 leaf. (**A**–**I**): Transmission electron (TEM) images of the phloem in noninfected (NI) (**A**–**C**) and infected (I) (**D**–**I**) plants. Images are representative of the sieve elements observed, with *n* = 11–29 for healthy plants and *n* = 63–73 for infected ones, with 58 SE in total observed for healthy and 200 SE in total for infected plants. (**A**,**D**,**G**) WT, (**B**,**E**,**H**) *SUT1*-AS plants and (**C**,**F**,**I**) *SUT2*-AS plants. (**A**–**F**) Transversal sections and (**G**–**I**) longitudinal sections. ^✱^ Phytoplasma, CC: companion cell, SE: sieve element, SP: sieve plate (white arrows in (**G**-**I**) and P-p: P-proteins (white arrows in (**D**)). Scale Bars, 2.5 µm. (**J**) SE cross-sectional areas in the phloem of not infected and infected plants, determined from TEM images (*n* = 8–22). Asterisks above the bars indicate significant differences by a *t*-test in *SUT1*- or *SUT2*-AS plants compared to WT plants in the same genotype. The effects due to the infection (Inf), genotype (G) and their interaction (G x Inf), determined using a two-way ANOVA, are reported above the plot (* *p* < 0.05 and *** *p* < 0.001; ns, not significant.

We investigated the anatomy of the vascular tissues in L1 leaves, since hypertrophy of the vascular parenchyma cells characterized a well-established Stolbur infection [42]. In noninfected plants, the histology of the midribs was similar, regardless of the genotype (Figure S1). In infected plants, no changes were observed either, regardless of the genotype: neither phloem nor xylem hyperplasia was detected. Looking at the ultrastructure of the phloem cells, we imaged at medium and high magnification 58 SE from healthy plants and 200 SE from infected plants (Figure 3A–I). Bacteria were visible in the SEs of infected plants. No differences were noticed in the midrib histology of L1 leaves when comparing the WT and AS lines in either the control or infected plants. Callose deposits in the SEs did not differ between healthy and infected plants, irrespective of the genotype. At a cell level, the average cross-sectional area of the SEs was less in *SUT1-* and *SUT2*-AS plants than in WT, with infection having a small additional effect. The SE area reduced to 50% or 40% of WT for *SUT1-*AS and to 65% and 55% of WT for *SUT2*-AS in healthy and infected plants, respectively (Figure 3J).

**Figure 4:**
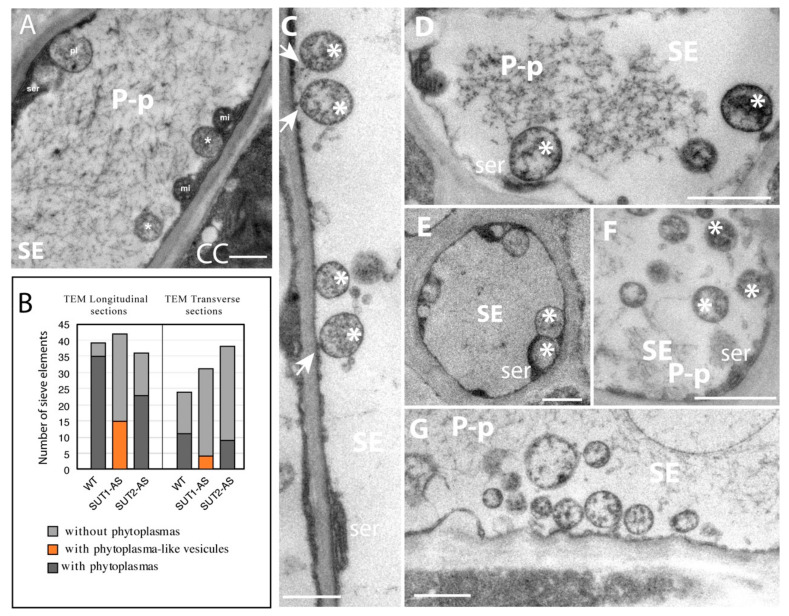
Frequency and location of phytoplasmas in the sieve elements of infected plants. (**A**,**C**–**G**) Details of TEM micrographs in the SE of infected main veins of L1 (leaflets 4 or 5) showing the location of parietal phytoplasma. (**B**) Frequency of SE with phytoplasmas. (**A**) Distinctive features of the phytoplasma observed with TEM compared to plastids and mitochondria on a SE longitudinal section. A mature sieve–tube plastid (pl), around 1-µm-wide, exhibits a sparse stroma enclosing a dense inclusion of a proteinaceous type. Phytoplasma (asterisks), less wide, display a loose fibrillar content, whereas the mitochondria matrix (mi) is dense, with clearer cristae. (**C**,**G**) TEM images of the WT (**C**,**D**), *SUT1-*AS (**E**,**F**) and *SUT2-*AS lines (**G**). In (**C**), white arrows indicate attachments of phytoplasma to the SE plasma membrane. * Phytoplasma, SE: sieve element, P-p: filamentous P-proteins and ser: SE reticulum. ser: sieve element reticulum. Bar: 1 µm. (**B**) Number of SEs with or without phytoplasmas observed in the phloem of infected L1 leaves. The data were determined with TEM images of transverse or longitudinal sections of the phloem of WT, *SUT1*-AS and *SUT2*-AS infected plants. A total of 63, 73 and 74 SEs were imaged for WT, *SUT1*-AS and *SUT2*-AS plants, respectively. Phytoplasmas were unambiguously identified in the SEs of WT and *SUT2*-AS plants (5–30 phytoplasmas per cell). In *SUT1*-AS plants, no typical phytoplasma were observed, but phytoplasma-like vesicles were observed, less dense and looser.

Phytoplasma, recognizable by their round-shaped bodies enclosing DNA strands and granular ribosomes (Figure 4A), were abundant in the SEs of WT (Figure 3D,G) and *SUT2*-AS plants (Figure 3F,I), observed in most SE sections (Figure 4B). Very few typical phytoplasma were observed in the SEs of infected *SUT1*-AS plants (Figure 3E,H and Figure 4B), consistent with the low rRNA (Figure 2F) and, in which, we rather observed phytoplasma-like vesicles looser than regular phytoplasmas. Phytoplasma were located either in the lumen or at a parietal location (Figure 4C–G), although no reorganization of the plasma membrane and sieve element reticulum was observed in contrast to later stages [43]. We also observed contacts between the plasma membranes and parietal phytoplasmas (Figure 4C,D,F), with, in some cases, embedding of phytoplasmas by the sieve element reticulum (Figure 4E).

**Figure 5:**
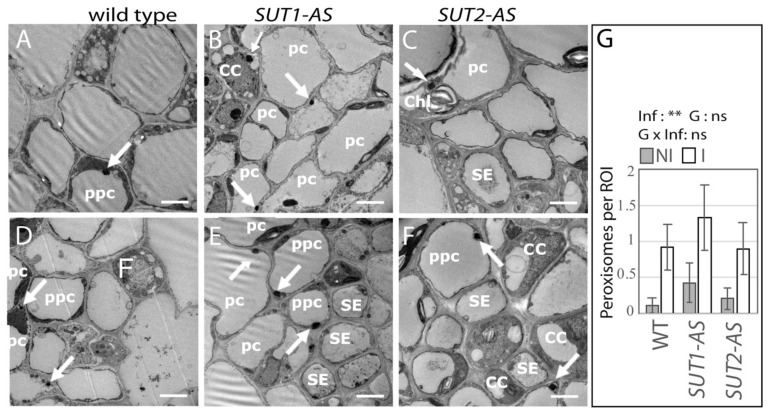
Frequency of peroxisomes in the phloem of infected and noninfected tomato plants. (**A**–**F**) TEM images of the main vein phloem cells in leaf L1 in not infected (**A**–**C**) and infected (**D**–**F**) plants. (**A**,**D**) Wild-type (WT), (**B**,**E**) *SUT1*-AS and (**C**,**F**) *SUT2*-AS transversal sections showing the location of peroxisomes (white arrows in (**A**–**F**)) in phloem cells. Peroxisomes, easily recognizable by their large crystals, were found in parenchyma cells (pc), inside or close to the phloem bundle in the three lines, and were rarely observed in other phloem cell types, such as in companion cells (in (**B**)). CC: companion cell, SE: sieve element, pc: parenchyma cell, ppc: phloem parenchyma cell and Chl: chloroplast. Bars, 2.5 µm. (**G**) The histogram shows the average number of peroxisomes per ROI (+/− se), with *n* = 6–14. ROI: region of interest, NI: not infected and I: infected, ** *p* < 0.01.

Peroxisomes, recognizable by their typical crystals, were observed in phloem parenchyma cells and at the periphery of the vascular bundles (Figure 5A–F), with a higher frequency in infected plants, with one peroxisome per region of interest (ROI) in infected plants compared to 0.2 in noninfected plants (*p* = 0.002, Figure 5G).

### 2.3. Leaf Sugar and Starch Content in Response to the Infection

Since phytoplasma infection can lead to phloem occlusions and impair photoassimilate translocation in host plants, we analyzed the sugar and starch contents in the lamina of L1, L4 and L6 leaves of noninfected and infected plants (Figure S2). *SUT1*-AS plants showed higher glucose and fructose contents in the L6 leaves compared to WT plants, which is consistent with previous reports on tomato plants [55]. Surprisingly, there was little effect of the infection, except for a higher starch content in L1 leaves of all three genotypes, and a subsequent lower sucrose-to-starch ratio. No effect was observed on the hexose-to-sucrose ratios in L1 and L4 leaves, confirming that there was little variation in the steady-state level of soluble sugars in WT and AS lines, regardless of infection.

### 2.4. Infection Impairs Phloem Exudation of Sugars and Organic Acids

**Figure 6:**
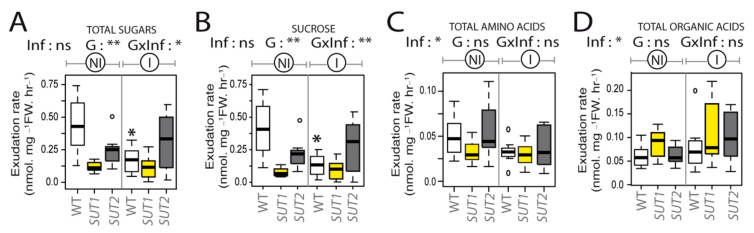
Rate of phloem exudation of metabolites from L3 leaves in response to the infection. Exudation rate is expressed in nmol mg^−1^ fresh weight (FW) per hour of exudation. Boxplots show rates of total sugars (**A**), sucrose (**B**), total amino acids (**C**) and total organic acids (**D**) in noninfected (NI) and infected plants (I). The probabilities obtained by a two-way ANOVA, indicating the effects of the Infection (Inf), the Genotype (G) and interaction of Genotype by Infection (GxI), are shown on each boxplot header. The box and whisker plots show the distribution of the biological replicates. Inside black lines represent medians, top and bottom ends of the boxes represent the first and the third quartiles, respectively, and whisker extremities (open circles) represent the maximum and minimum data points when different from the first and third quartiles (*n* = 6–8). Asterisks above whisker plots indicate significant differences by a *t*-test in infected compared to the noninfected plants of the same genotype. *p*-values: * *p* < 0.05 and ** *p* < 0.01; ns, not significant.

Even if there was no effect of phytoplasma on leaf sugar homeostasis in mature L4 and L6 leaves, we measured in noninfected and infected plants the rate of phloem sugar exudation. It was measured on the L3 leaf, which was similar to the L4 leaf for leaf expansion, both being source leaves (Figure 6). The collect of phloem exudates was done by EDTA-facilitated exudation, a method that has been successfully applied in tomatoes to analyze phloem sap amino acids profiles and phloem-soluble carbohydrate flows [57,58]. We observed a major genotypic effect on the sugar and sucrose exudation rates in noninfected plants (Figure 6A,B), indicating that the disruption of *SUT1* and *SUT2* impaired the phloem sugar release (ANOVA; *p* ≤ 0.01), with an exudation rate for *SUT1*-AS plants reducing to 27% of that for WT plants and to 56% for *SUT2-*AS plants. The L3 leaves of infected WT plants had a lower exudation rate of sugars and sucrose (38% of noninfected plants). The infection of the AS plants caused no further significant reduction than the effect of the transporter disruption.

Amino acids and organic acids were also measured in the phloem sap-enriched exudates of noninfected and infected plants (Table S2). In exudates, the most abundant amino acids were glutamine, serine, asparagine, alanine and the nonproteinogenic GABA. The most abundant organic acids were malate, glycolate and glyoxylate (Table S2). No genotypic effect was observed for the exudation rates of amino acids and organic acids (Figure 6C,D), but the ANOVA showed a reduction of the exudation rate of amino acids in infected plants (Figure 6C). An opposite effect was observed on the exudation rates of organic acid, with higher values in infected plants compared to noninfected ones (Figure 6D).

### 2.5. Metabolite Content of Phloem Sap-Enriched Exudates

**Figure 7:**
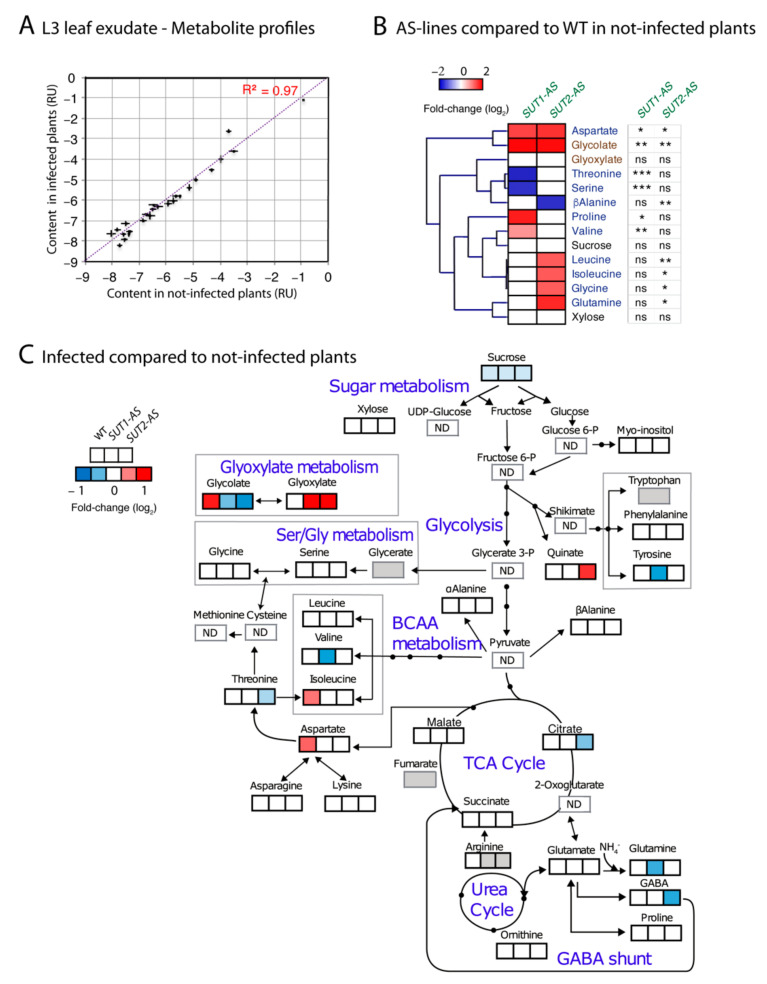
Comparison of metabolite profiles of the phloem sap-enriched exudate from the L3 leaf of not infected and infected plants. (**A**,**B**,**C**) Analysis of the metabolite content determined on the exudate of the third leaf for WT, *SUT1-* and *SUT2*-AS lines. (**A**) Pairwise comparisons and *R*^2^ correlation coefficients between metabolite profiles in infected and not infected plants in the 3 genotypes. The plots show for each metabolite its contents in the exudates of not infected plants (*X*-axis) and infected plants (*Y*-axis). The linear regression indicates that most metabolites remained stable in both conditions. (**B**) Heat map showing significant fold changes in metabolite contents in the phloem sap-enriched exudates from the L3 leaves of not infected *SUT1*- and *SUT2*-AS plants compared to not infected WT plants (*n* = 7–8). Values are shown in a blue-to-red log_2_ scale, with blue for negative values, red for positive values and white for no difference. On the right panel: significance of the effects due to genotype (G), determined using one-way ANOVA (* *p* < 0.05, ** *p* < 0.01 and *** *p* < 0.001; ns, not significant). (**C**) Heat map showing significant fold changes in the contents of metabolites in phloem sap-enriched exudates in response to infection (*p*-value < 0.05 on a paired *t*-test). From the left to the right sides, responses in wild-type (WT) on the left side, *SUT1-*AS line in the middle and *SUT2*-AS line on the right side. Values are shown in a blue-to-red log_2_ scale, with blue values for metabolites showing a smaller content (and red values for higher) due to infection. BCAA: branched-chain amino acids. TCA: tricarboxylic acid cycle. In white: nonsignificant variations. In grey: missing values.

The lower sucrose exudation rate observed for infected WT plants could result from sugar consumption by bacteria from the cleavage of sucrose in the SEs to provide precursors for the synthesis of callose or cell wall precursors and an increased resistance to or from a wound reaction, which reduces the phloem capacity. To identify, within the metabolite profiles of the phloem sap-enriched exudates, specific differences in their proportions, independent of the exudation rate, the profiles were adjusted using a method of normalization that has been developed for the analysis of phloem sap-enriched exudates [59]. The normalized values, termed “content”, showed high positive correlations between infected and noninfected metabolite profiles (correlation coefficient (*R*^2^) > 0.97; Figure 7A), revealing a strong homeostasis in phloem sap-enriched exudate composition. In noninfected plants, there was no modification of the sucrose content in the L3 exudates of the three genotypes, yet the glycolate content was higher (Figure 7B). The contents of the branched amino acids (valine, leucine and isoleucine); aspartate; glutamine; proline; serine and glycine also varied with the genotype (Figure 7B). In response to phytoplasma infection, the contents of most metabolites were not altered (Table S3 and Figure 7C). Overall, no correlation was observed between the contents of the exudate metabolites and the average bacterial rRNA accumulation quantified in the L1 and L4 leaves. The malate content, an abundant organic acid, showed no variation.

**Figure 8:**
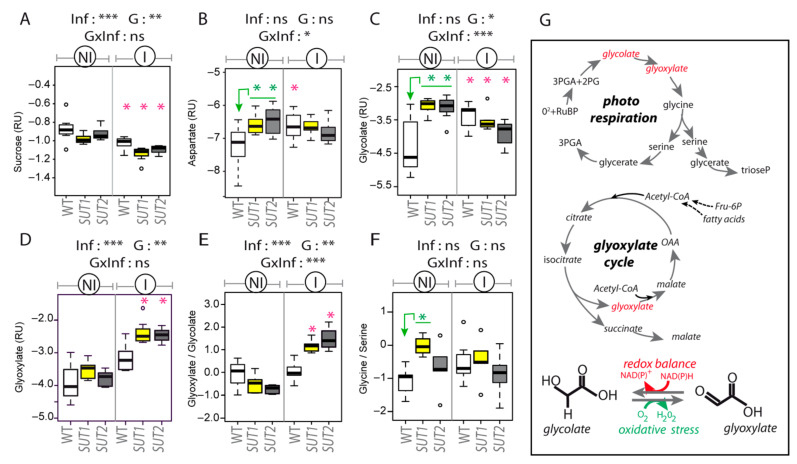
Main variations in the metabolite contents of phloem sap-enriched exudates in response to the infection. (A-F) Boxplots with the contents in the exudates from the L3 leaf of (**A**) sucrose, (**B**) aspartate, (**C**) glycolate and (**D**) glyoxylate and the content ratios for (**E**) glyoxylate-to-glycolate and (**F**) glycine-to-serine. Noninfected plants: NI, Infected plants: I and RU: relative units for content, plotted on a log_2_ scale. The box and whisker plots show the distribution of the biological replicates. Inside black lines represent medians, top and bottom ends of the boxes represent the first and the third quartiles, respectively, and whisker extremities (open circles) represent the maximum and minimum data points when different from the first and third quartiles (*n* = 6–8). Above each boxplot, the significance of the effects due to the infection (Inf), genotype (G) and their interaction (G × Inf) (* *p* < 0.05, ** *p* < 0.01 and *** *p* < 0.001; ns, not significant). Inside boxplots in green, *t*-test comparing AS lines with WT for NI plants and, in red, *t*-test comparing I and NI plants for each genotype. (**G**) Glyoxylate can be produced either via photorespiration or via the glyoxylate cycle, the latter being a bypass of the TCA cycle. Glyoxylate and glycolate are reversibly converted by glyoxylate reductases (GLYR) and glycolate oxidases (GOX), reactions that are controlled by the redox status and contribute to the production of reactive oxygen species (ROS) and the conversion of NAD(P)H into NAD(P)+. 2-PG: 2 phosphoglycolate, 3-PGA: 3 phosphoglycerate and RuBP: ribulose 1,5-bisphosphate.

Nevertheless, irrespective of genotype, infection slightly reduced the content of sucrose (10% less compared to noninfected) (Figure 8A and Table S3). Infection increased the content of glycolate and aspartate in WT plants (approximately 80% and 50% more, respectively). By contrast, we observed in the AS genotypes no change in the aspartate content, a lower glycolate content and a higher glyoxylate (approximately two-fold increase) (Figure 8B,C and Table S3), with a higher value for the glyoxylate-to-glycolate ratio in the AS lines in infected plants compared to noninfected ones, which was confirmed by the strong effect of the interaction “infection per genotype” on this ratio and on the aspartate and glycolate contents (Figure 8B,C,E). Since a higher accumulation of glycolate and glyoxylate may be indicative of an oxidative stress, we determined the glycine-to-serine ratio, a marker of photorespiration [60], a pathway frequently upregulated under stress conditions for protecting against oxidative damages and consuming excess reductants [61]. This ratio was higher in the *SUT1*-AS plants compared to the WT plants, a response that was not observed in the other AS line. This difference was highly significant in the noninfected plants (*p* = 0.002).

### 2.6. Transcriptional Reprogramming of Selected Genes

**Figure 9:**
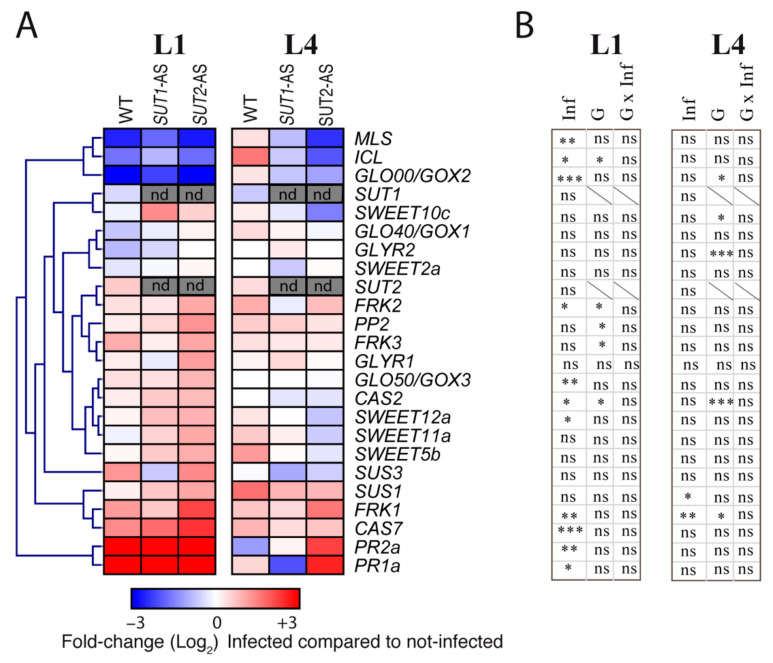
Transcript profiling of candidate genes in infected plants. (**A**) Inserted heat maps showing fold changes between infected and noninfected plants in each genotype in L1 and L4 leaves (*n* = 4). Fold changes were determined after normalization by the reference genes, and values are shown on a log_2_ scale, with blue values for metabolites showing a smaller content due to infection and red values for a higher content. In grey, not determined (nd). (**B**) Results of the two-way ANOVA for each gene, with the effects of infection (Inf), genotype (G) and interaction (G × Inf). *p*-values: * *p* < 0.05, ** *p* < 0.01 and *** *p* < 0.001. ns, not significant.

The above-mentioned hypotheses were further investigated by the analysis in L1, L4 and L6 leaf midribs of the expression of genes encoding either stress markers or involved either in the glyoxylate cycle, photorespiration or sugar transport and metabolism (Table S4). Selected genes for glyoxylate cycle and photorespiration were coding for glycolate oxidases (*GLO00/GOX2*, *GLO40/GOX1* and *GLO50/GOX3*); glyoxylate reductases (*GLYR1* and *GLYR2*); isocitrate lyase (*ICL*) and malate synthase (*MLS*). Genes coding for callose synthases (*CAS2* and *CAS7*) and acidic pathogenesis-related (PR) proteins that are hallmarks of SA-mediated defenses (*PR1a* and *PR2a*) were included as stress markers. *CAS7* was reported to be the only callose synthase gene, out of eight retrieved from the NCBI database, to show an increased expression in symptomatic tomato leaves in comparison to noninfected ones [42]. For sugar transport and metabolism, we included genes coding for fructokinases (*FRK1*, *FRK2* and *FRK3*); sucrose synthases (*SUS1* and *SUS3*) and *SWEET* sugar facilitators (*SWEET2a*, *SWEET5b*, *SWEET10c*, *SWEET11a* and *SWEET12a*). A gene coding for a phloem marker (*Phloem protein 2, PP2*) was added as well. The expression of most genes varied depending on the leaf (Table S5), so the responses were analyzed per leaf level. A strong correlation (*R* > 0.7, *p* < 0.001) was found between the expression of *CAS7*, *PP2* and *FRK3* (Table S6). In noninfected plants, differences were observed in the transcript levels for *FRK2* and *FRK3* in L1 leaves and for *GLO00*/*GOX2* and *GLYR2* in the L4 leaves in *SUT1*- and *SUT2*-AS plants, compared to WT plants (Figure S3). The data indicated that the downregulation of *SUT1* and *SUT2* affected the sugar metabolism and photorespiration, depending on the leaf level. In infected plants, all L1 leaves showed symptoms, and, correspondingly, infection raised the *PR1a* and *PR2a* transcript levels in all genotypes (Figure 9).

Higher transcript levels were also observed for *CAS7*. We observed no significant response in L1 leaves on the expression of *SUT1*, *SUT2*, *SWEET2a*, *SWEET11a*, *SWEET5b* and *SWEET11c*. By contrast, the transcript levels for *GLO50/GOX3*, *SWEET12a*, *FRK1* and *FRK2* were higher and lower for *GLO00/GOX2*, *ICL* and *MLS* (Figure 9). The effects of infection were lower in L4 than L1 (Figure 9), with higher transcript levels of *SUS1* and *FRK1*. Interestingly, no significant changes were observed in the L4 and L1 leaves for the transcription levels of *SUT1* and *SUT2* in infected WT plants compared to noninfected. No correlation was observed between the accumulation of *PR1a*, *PR2a*, *CAS2* and *CAS7* and the accumulation of phytoplasma rRNA in L1 and L4 leaves. Remarkably, we observed several correlations between the expression of *FRK1*, *FRK3*, *SUS1* and *SUS3* and the expression of *ICL*, *MLS*, *GOX1*, *GOX2*, *GOX3* and *GLYR1* (Table S6). A negative correlation was found between the expressions of *FRK1* and *ICL* and *GLYR2* (*p* < 0.001), while a positive correlation was found between the expression of *SUS1*, *SUS3* and *FRK3* and the expression of *GOX1*, *GOX3* and *GLYR1* (*R* > 0.44, *p* < 0.001) (Table S6).

## 3. Discussion

### 3.1. SUT1-AS and SUT2-AS Lines as Tools to Study Coupling between Sugar Transport and Metabolism

Phloem loading and the equilibrium of the release/retrieval of sucrose are expected to be altered in *SUT1-* and *SUT2*-AS lines. Our data show that the sugar exudation rate from excised leaves was lower in these lines compared to WT, with a marked effect in *SUT1*-AS plants. Interestingly, in these AS lines, the cross-sectional area of SEs in the midribs of apical leaves was reduced compared to WT. The phloem mass flow, being convective, is the product of flow velocity, sap concentration and cross-sectional area of functional SEs, so the lower sucrose transport in the AS lines could also be due to the reduced SE cross-sectional area in addition to a decrease in flow velocity. Interestingly, the sucrose content was not impaired in the exudate of these lines, revealing a tight homeostasis in the phloem exudate composition. These findings are consistent with early reports of the tight homeostasis of sucrose concentrations and sap osmotic potentials in *Sonchus oleraceus* during phloem pathway blockage [62].

Interestingly, higher aspartate and glycolate contents were observed in the exudates of the AS lines compared to WT, indicating that the impairment of sugar transport induces changes in amino acids and organic acids exudate contents. Aspartate is abundant in the phloem sap of many species, including tomatoes and potatoes [57,63], participating in nitrogen remobilization. A higher aspartate content suggests that the impairment of phloem sugar transport alters, likely in the phloem tissue, the nitrogen assimilation or remobilization. In contrast, little is known on the synthesis and translocation of glycolate in the phloem. In the AS lines, the upregulation of *GOX2* and downregulation of *GLYR2* and the higher glycolate content in the sap indicate that the impairment of phloem sugar transport leads to changes in glycolate–glyoxylate metabolism.

### 3.2. Reduced Phloem Flow in Infected Plants

**Figure 10:**
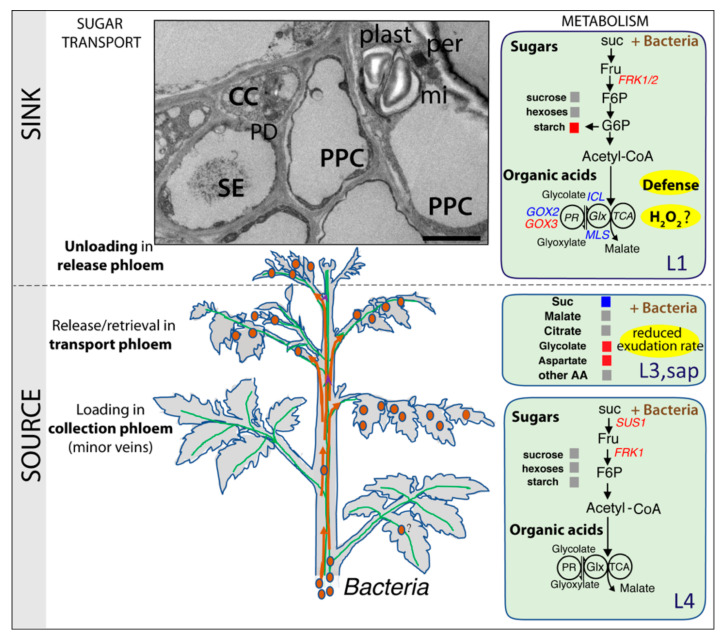
A model of our inferred responses in the young (sink) and mature (source) leaves of a tomato infected plants. On the right side, primary metabolic steps regulating the levels of sugars and organic acids (METABOLISM) in L1 and L4 leaves. Upregulated genes are shown in red, and downregulated genes are shown in blue. The metabolites in exudate from L3 are shown in grey (no change), blue (decrease) or red (increase). Similarly, the soluble sugars and starch contents are shown in the L1 and L4 leaves. The TEM inset shows the main phloem cell types: CC: companion cells, SE: sieve elements, PPC: phloem parenchyma cells, plast: plastid, mi: mitochondria, per: peroxisome and PD: plasmodesmata, with typically close locations of the peroxisome with mitochondria and plastids with starch granules (Bar: 2.5 µm). Suc: sucrose, Fru: fructose, F6P: fructose-6-phosphate, Glc: glucose, G6P: glucose-6-phosphate, AA: amino acids, Glx: glyoxylate cycle and PR: photorespiration.

In infected plants, we measured the sugar exudation rate to reflect sucrose loading and export from a leaf before excision, despite any concerns arising from excision and contamination during phloem sap collection. We observed a reduction of the sugar exudation rate to one-third of the level observed in noninfected plants (Figure 10), which is consistent with the reduction in phloem flow observed in phytoplasma-infected Arabidopsis [6]. A reduction in sap flow, rather than a reduction of sucrose concentration of the sap itself, is likely, since the sap composition changed rather little. SE occlusion by callose deposition could be a reason for this reduction in infected plants, a hypothesis frequently proposed [12,42,64]. However, if the reduction in flow was due to the occlusion of the sieve pores, a similar response is expected to be observed in the antisense lines, which was not the case, the infection hardly affecting exudation for the *SUT1*-AS and *SUT2*-AS plants. Alternatively, the infection could have altered the sugar loading and transport, which may be associated with increased release in surrounding tissues. Interestingly, because we also observed in the AS lines high glycolate and aspartate contents in the exudates associated with impaired sugar transport, one hypothesis is that higher glycolate and aspartate levels in the exudates of infected WT plants are due to impaired SUT1 and/or SUT2 functions, leading to the decline in phloem flow and/or changes in release/retrieval balance. An increase of sugar delivery to lateral tissues in the infected phloem tissues could be required for the higher demand of carbon skeleton necessary for the vascular hyperplasia that is associated with well-established Stolbur infection in tomatoes [42].

### 3.3. Glycolate–Glyoxylate Metabolism in Symptomatic Leaves

Several reports indicate that a phytoplasma infection triggers oxidative stress in symptomatic leaves. For example, high levels of H_2_O_2_ and ROS were found in the symptomatic leaves of *Morus multicaulis*, *Pennisetum purpureum* and *Ziziphus jujuba* infected by phytoplasma [25,26,27]. Peroxisomes are important sites of production of H_2_O_2_ and other ROS, generated by different metabolic pathways. One of them, the glyoxylate cycle, can be induced when plants respond to pathogens and phloem-feeding insects [65,66,67,68]. Interestingly, in the L1 leaves of infected tomato plants, more peroxisomes were observed in the symptomatic leaves, revealing that the peroxisome biogenesis was altered. The deregulation of several genes involved in the glyoxylate cycle or photorespiration (*MLS*, *ICL*, *GOX2* and *GOX3*) in infected plants also indicate that the infection modulates the glycolate–glyoxylate metabolism in symptomatic leaves (Figure 10). These changes in glycolate–glyoxylate metabolism in phytoplasma-infected leaves could generate H_2_0_2_ and ROS in the symptomatic leaves and trigger a defense response in these leaves, supported by the upregulation of *PR1a* and *PR2a*, both markers of the salicylic acid (SA)-signaling pathway.

### 3.4. Sugar Metabolism in Symptomatic Leaves and Vascular Hyperplasia in Infected Plants

The symptoms were also associated, one day after their appearance, with an increase in starch accumulation, which could be the consequence of the reduction in leaf growth. Our data show, in the symptomatic leaves, an upregulation of *FRK1* and *FRK2*, indicating that the infection alters glycolysis, with the potential consequence of higher starch accumulation. Both genes are expressed in the vascular cells [69] and are proposed to play a role in the supply of carbon for starch accumulation [54,70]. Interestingly, fructose phosphorylation by FRK2 has also been shown to be important for callose deposition in tomatoes [71], so our findings for symptomatic leaves may be related to the upregulation of *CAS7* and callose deposits [42]. Alternatively, the upregulation of *CAS7* may be related to phloem hyperplasia, which is observed at the more advanced stages of infections [42]. Tomato *CAS7* is orthologous to Arabidopsis *CalS7*, a gene expressed in the phloem and necessary for phloem development [72,73]. This hypothesis is supported by the observation of a correlation between the expression of *CAS7* with that of *FRK3* and *PP2*, both genes being also specifically expressed in the vascular tissues in tomatoes [42,74].

### 3.5. Homeostasis of the Metabolite Content in Phloem Exudates of Infected Plants

Our metabolomic survey of phloem exudates indicates that the infection triggers very few changes in the overall phloem sap composition, as described in phytoplasma-infected *Prunus* species [35] but in contrast to what was described in exudates collected from symptomatic leaves of phytoplasma-infected mulberries [25]. The novelty of our study compared to previous ones was to study separately the general effect of infection on the sap flow from that on the relative composition of each of the transported metabolites. This allowed us to analyze more precisely the variations in the contents of individual metabolites. Some of the effects described in previous studies may primarily reflect the overall effects on phloem flow rather than variations in the composition.

In Stolbur-infected tomatoes, the infection leads to a slight decline in the exudate sucrose content, regardless of the genotype (about 10% reduction), suggesting that it was independent of the *SUT1* and *SUT2* functions. It may result from a higher consumption of sucrose due to phytoplasma in SEs, a cleavage of sucrose to provide precursors for the synthesis of callose or lower sugar loading or retrieval along the pathway. Phytoplasma, having small genomes, are auxotrophs for many nutrients, with malate being potentially a main source of carbon [5,75,76]. Interestingly, our data indicates that the infection did not affect the malate contents in phloem sap-enriched exudates. They differ from other reports of higher accumulations in malate reported in the sap and in the main veins of infected plants [25,33,36]. Interestingly, malate accumulates in the apoplasm [77,78]. The close abutting of phytoplasma to the SE membranes at an early stage (Figure 4), as reported at later stages [43,44], could further indicate that nutrients such as malate are taken from the apoplasm through connections with SE membranes.

### 3.6. Altered Susceptibility to the Infection in the SUT1-AS

The severity of the symptoms was identical in WT and in the *SUT2*-AS line, although bacteria multiplication appeared to be a little higher in the case of the *SUT2*-AS line. Remarkably, the *SUT1*-AS line was less susceptible to the infection compared to the other genotypes. It could be the consequence of metabolic changes, supported by changes in the gene expression, such as *FRK3*, to such an extent that defense cascades are being triggered more efficiently. Interestingly, in the exudate of noninfected *SUT1*-AS plants, we observed a high glycine/serine ratio, which may indicate elevated photorespiration and more proline, whose concentration in the phloem sap can increase under stress conditions [79,80,81]. The upregulation of *GOX2* in the mature leaves of *SUT1*-AS plants may be related to an oxidative stress in the phloem of the *SUT1*-AS line. Interestingly, a high synthesis of H_2_O_2_ has been associated with recovery from phytoplasma-associated disease in grapevines [82,83]. More work needs to be done to determine whether oxidative stress and/or antioxidant defense systems are triggered in the *SUT1*-AS line and can be detrimental to phytoplasma infection.

Interestingly, the first symptoms appeared in all lines at the same day, arguing that variations in the infection may not solely be due to differences in the kinetics of propagation along the plant. The number of bacteria units delivered into the translocation stream might be reduced, which could explain the lesser susceptibility in the *SUT1*-AS plants and very few bacteria in the SE of their L1 leaves. However, the speed of spreading of the phytoplasma are too low for the movement to be simple convection in the flow, which suggests that the bacteria are stationary in the SEs throughout part of their lifetimes, possibly anchored to the membranes. Our findings could support the notion of a restriction of passive translocation due to the retainment of bacteria in source leaves, a scenario that was also supported by a report on the phytoplasma colonization of *Euphorbia pulcherrima* [9]. Whether oxidative stress influences the retainment of phytoplasma and limit their translocation is not known.

Since phloem sugar transport has seasonal variations, our observations could help to explain the seasonal fluctuations in the colonization of fruit trees by phytoplasmas [10]. In addition, environmental clues, such as mineral deficiency, water deficit, low temperatures or variations in light intensity, can modify phloem sugar transport [84], with stress conditions leading, in some cases, to higher carbon allocation to sink organs [85]. These observations indicate that agricultural practices reducing mass flow in field conditions may be a way to limit the multiplication of phytoplasma and control disease development, with a need to balance the benefits against lost production.

### 3.7. Concluding Remarks

A detailed analysis of the effects of infection at the onset of symptoms reveals that major changes already occurred in sugar transport, characterized by a reduction in the rate of exudation in asymptomatic source leaves and an increase in the aspartate and glycolate contents in the exudates (Figure 10). By comparing this with what occurs in antisense lines in which the expression of *SUT1* and *SUT2* is altered, the data suggests that a phytoplasma infection compromises the function of *SUT1* and *SUT2*. Furthermore, phytoplasma multiplication is associated with the impairment of glycolysis and peroxisome metabolism according to the leaf levels. In infected, asymptomatic source leaves, the deregulation of *SUS1* and *FRK1* indicates an alteration of the sugar homeostasis, which may be related to the impairment of sugar transport (Figure 10). In symptomatic sink leaves, higher starch levels and the upregulation of genes associated with SA-mediated defenses could be related to an alteration of photorespiration and the glyoxylate cycle, as suggested by the altered expression of genes acting in these metabolic pathways. Finally, the correlation observed between the transcript profiles of *CAS7* with that of *FRK3* and *PP2* indicates that the CAS7 response may be associated with the hyperplasia of vascular tissues observed at more advanced stages of infection.

## 4. Materials and Methods

### 4.1. Plant Material and Infection by Phytoplasma

Tomato plants (*Solanum lycopersicum* L., cv. Money Maker) were grown in a glasshouse (27/20 °C day/night in a 16-h photoperiod) in soil with sand and organic matter (20:80). Seeds of the antisense (AS) lines *SUT1*-15 (hereafter called *SUT1*) and *SUT2*-12 (called *SUT2*) [55] and wild-type Money Maker plants (WT) were received from the Biology Department in Humboldt University of Berlin (Germany). AS lines showed a slower growth at the beginning of their development compared to WT, and sowing of AS plants was done 2.5 weeks earlier than WT to obtain at grafting time the same height of the plants and number of expanded leaves (7 fully expanded). Eight and ten-and-a-half weeks after sowing, respectively, for the WT and *SUT1*-AS and *SUT2*-AS lines, inoculation was performed by chip grafting [41] with the PO strain of Stolbur phytoplasma (STOL-PO), a strain isolated from Pyrénées Orientales in South France [41] and belonging to the “*Candidatus* Phytoplasma solani” species [40], and with a scion from infected WT tomato plants. Noninfected controls were grafted with noninfected scions. For each genotype, 8 plants were grafted with, respectively, infected and noninfected materials for a total of 48 plants. The first visible symptoms of infection appeared at 17 days after grafting (DAG). At this stage, six new leaves emerged on the grafted plants, irrespective of the genotype (Figure 1). Sampling was done one day after, at 18 DAG. For all analyses, plant samples were collected between 11:00 h and 12:00 h, two hours before the middle of the day.

The severity of phytoplasma symptoms was recorded at 18, 24 and 27 DAG by a notation scale from 0 to 4, with class 0: no symptoms, class 1: beginning of curling of the leaflets, class 2: mild curling of leaflets, class 3: light yellowing of interveinal tissue of the leaflets and leaflet deformation, class 3.5: interveinal yellowing and severe curling and class 4: severe reduction of the leaflet’s area, with leaflet chlorosis and a crooked shape, a characteristic of Stolbur disease on tomato [86].

### 4.2. Identification of Tomato Gene Sequences and Design of Primers

Tomato sequences were retrieved from the NCBI (http://www.ncbi.nlm.nih.gov/) and Phytozome (https://phytozome.jgi.doe.gov/pz/portal.html) databases [87], and the expression pattern was analyzed on the Bio-Analytic Resource [88] (http://bar.utoronto.ca/efp_tomato/cgi-bin/efpWeb.cgi). Information about selected genes is reported in Supplementary Table S4, and the primers used in real-time qPCR experiments are listed in Supplementary Table S7. The efficiency of each primer couple was evaluated as described in Pfaffl [89].

### 4.3. Total RNA Extraction and Plant Gene Expression

Material for RNA extraction was sampled from leaves L1, L4 and L6 (Figure 1). The three leaves were sampled on each plant, with four plants (biological replicates) sampled for each genotype (WT, *SUT1*-AS and *SUT2*-AS lines) and for the two conditions (noninfected and infected). The midrib of the leaflets 1, 2 and 3 were collected and frozen in liquid nitrogen. Total RNA was isolated following a TRIzol-based extraction (Invitrogen, Thermo Fisher Scientific, Waltham, MA, USA) and a DNase treatment (DNase I RNase-free; Thermo Fisher Scientific, Waltham, MA, USA). The first strand of cDNAs was synthesized with M-MLV reverse transcriptase (Thermo Fisher Scientific, Waltham, MA, USA) starting from 1 µg of total RNA. Real-time qPCRs were performed on a CFX96 Real-Time PCR Detection System (Bio-Rad Laboratories, Hercules, CA, USA) using Takyon ROX SYBR 2X Master Mix dTTP blue (Eurogentec, Seraing, Belgium), beginning with a step at 95 °C for 3 min, followed by 40 cycles for 15 s at 95 °C, 60 s at 60 °C and 30 s at 72 °C. The mean normalized expression (MNE) was calculated by the method of normalization described in [90] using *UBI*, *UPL3*, *PGK* and *UrK* as the reference genes and taking into account each primer couple efficiency (Table S7). Normalized data are expressed in relative units.

### 4.4. Phytoplasma Detection

Genomic DNA was extracted from 0.5 g of petiole and rachis from the leaflet 1 of the L1 leaf, using the Cetyl Trimethyl Ammonium Bromide method and 4 plants for each genotype and condition [91]. Two hundred nanograms of total leaf DNA were analyzed by real-time qPCR using primers for the *Methionine aminopeptidase* (*Map*) gene [92] (Table S7). Real-time qPCR was performed on a Light Cycler 480 (Roche) using SYBR^®^ Green master mix (Roche), imposing a step at 95 °C for 15 min, followed by 45 cycles for 15 s at 94 °C, 30 s at 62 °C and 30 s at 66 °C, then melting at 95 °C for 10 s and 66° for 10 s, continuous up to 95°. Absolute quantification was obtained with a DNA fragment from the *Map* gene cloned in pGEM^®^-T Easy vector (Promega, Madison, WI, USA), with 10 to 108 copies of the plasmid added on each PCR plate. rRNA abundance was analyzed to provide an additional index of phytoplasma multiplication. Using the same plants (4 plants for each genotype per condition), one ng of total RNA obtained from midribs of the L1 leaf (leaflets 1 to 3) was analyzed by real-time qPCR using specific 16S rRNA primers and expressed in relative units (Table S2).

### 4.5. Light Microscopy

Seventy micrometers-thick transversal sections of the midrib of leaflets 4 or 5 from the L1 leaf were cut using a vibratome (Leica) and stained with periodic acid (1% *w*/*v*, Sigma Aldrich, Saint Louis, MI, USA) and Schiff’s reagent (VWR, Radnor, PA, USA). Observations were carried out with an Axiozoom V16 macroscope (Zeiss, Oberkochen, Germany) equipped with a Plan-Neofluar Z 2.3x/0.57 RWD 10.6-mm objective. At least eight sections from four plants per genotype and condition were observed.

### 4.6. Ultrastructure Analysis Using Transmission Electron Microscopy

The midrib of leaflets 4 or 5 of the L1 leaf was examined by transmission electron microscopy (TEM). Samples were fixed in 2.5% glutaraldehyde–2% paraformaldehyde in 100-mM phosphate buffer, pH 7.2, for 3 h. They were post-fixed overnight at 4 °C with 1% osmium tetroxide, then dehydrated in a graded ethanol series and progressively infiltrated with Epon resin for 48 h. Curing occurred for 24 h at 60 °C. One hundred nanometer-thick sections were cut with an Ultracut S Microtome (Leica) and collected on hexagonal 600 mesh copper grids. Sections were observed at 120KV on a FEI Tecnai G2 Spirit TEM (FEI, Thermo Fisher Scientific, Waltham, MA, USA) equipped with an Eagle 4K digital camera (Fluid + Form by IconnTechs, Wan Chai, Hong Kong). Two plants per genotype were observed for infected plants and one for noninfected. For infected samples, both the transversal and longitudinal sections were analyzed. The number of peroxisomes in the phloem cells was counted per mesh (i.e., region of interest, ROI), one mesh corresponding approximately to 1000 µm^2^. Six–fourteen ROIs, focused on the phloem cells, including phloem parenchyma, phloem perivascular cells and companion cells, were observed per genotype and condition.

### 4.7. Sugars and Starch

Glucose, fructose and sucrose were quantified on the L1, L4 and L6 leaf levels. The leaf laminar tissues obtained after removal of the midribs of the leaflets 1, 2 and 3 were pooled, the midribs being used for RNA extraction (see above). Four plants were sampled for each genotype (WT, *SUT1*-AS and *SUT2*-AS lines) and for the two conditions (noninfected and infected). Sugar quantification was assayed enzymatically (Enzytec™ Sucrose/D-Glucose/D-Fructose-R-Biopharm AG kit, Pfungstadt, Germany) [93]. Starch quantification was determined after the release of glucose by incubation with a-amylase and amyloglucosidase (Sigma Aldrich, Saint Louis, MI, USA) [93]. Four replicates were analyzed per leaf level for each plant, with 4 plants for each genotype per condition. Leaf samples were collected between 11:00 h and 12:00 h.

### 4.8. Collection of Phloem Sap-Enriched Exudates

The apical leaflet 1 from L3 was used for phloem EDTA-facilitated exudation [94]. Immediately after cutting, the rachis of the leaflet was recut in 10-mM HEPES adjusted to pH 7.5 with NaOH and 10-mM Ethylenediaminetetraacetic acid (EDTA), pH 7.5, where it remained for 3–5 min. Then, the rachis was immersed in 400 µL of the same buffer and placed in a dark box with high humidity for 4 h for the exudation (from midday to 16:00 h). Tissue fresh weights (FW) were recorded at the end of the experiment to express the exudation rate per mg of FW. The exudates of 7 to 8 plants were analyzed for each genotype per infection condition. The exudates typically contained, on average, 95% sucrose relative to the total sugars, confirming enrichment in phloem sap (Figure S4).

### 4.9. Analysis of Phloem Sap-Enriched Exudates

Amino acids in exudates were analyzed in an UPLC-PDA system, as described in [95]. The quantification of sugars, sugar alcohols and organic acids was carried out using a GC-FID device [96]. To normalize the data, we determined a content for each metabolite within all metabolites in a sample (Figure S5), using the method originally developed for normalizing phloem exudates [59]. First, data were log_2_-transformed, and then, the value for each quantified metabolite in the profile was corrected with respect to the mean log content of all metabolites for the replicate, with a locally weighted scatterplot smoothing (LOWESS), all using R software (http://www.r-project.org). This normalization is required for the identification of the metabolites whose proportion is modified in response to the infection. ANOVA tests were performed on this dataset after removing any metabolite with missing values. A given metabolite’s content was declared different when the adjusted *p* after Benjamini-Hochberg correction was lower than 0.05.

### 4.10. Statistical Analysis

Statistical analyses on datasets, including ANOVA, were done using R statistical software. Correlations were calculated using the Pearson correlation coefficient and tested with the pairwise two-sided *p*-values and adjusted *p* determined with the Holm’s method. Hierarchical cluster analysis (HCA) graphical representation was done with Genesis version 1.7.6 (http://genome.tugraz.at/) after log_2_ transformation and normalization by the median, using the complete linkage clustering option and Euclidean distance.

## Data Availability

Not applicable, all data are provided in the ms.

## Supplementary Materials

The Supplementary Materials can be found at https://www.mdpi.com/1422-0067/22/2/745/s1.

## Abbreviations

SE	sieve element
CC	companion cell
ICL	isocitrate lyase
MLS	malate synthase
GLO	glycolate oxidase
FRK	fructokinase
GLYR	glycolate reductase
WT	wild type
AS	antisense
TCA	tricarboxylic acid cycle
SUT1	sucrose transporter 1
SUT2	sucrose transporter 2
EDTA	Ethylene Diamine Tetraacetic Acid
