# Supplementary material for: Involvement of SUT1 and SUT2 Sugar Transporters in the Impairment of Sugar Transport and Changes in Phloem Exudate Contents in Phytoplasma-Infected Plants
Source: Int J Mol Sci. 2021 Jan 13;22(2):745. doi: 10.3390/ijms22020745 (PMC7828548; doi:10.3390/ijms22020745)
Supplement: Supplementary file 1 [file ijms-22-00745-s001.pdf]

International Journal of
Molecular Sciences

MDPI

Supplementary Materials

Supplementary Figures

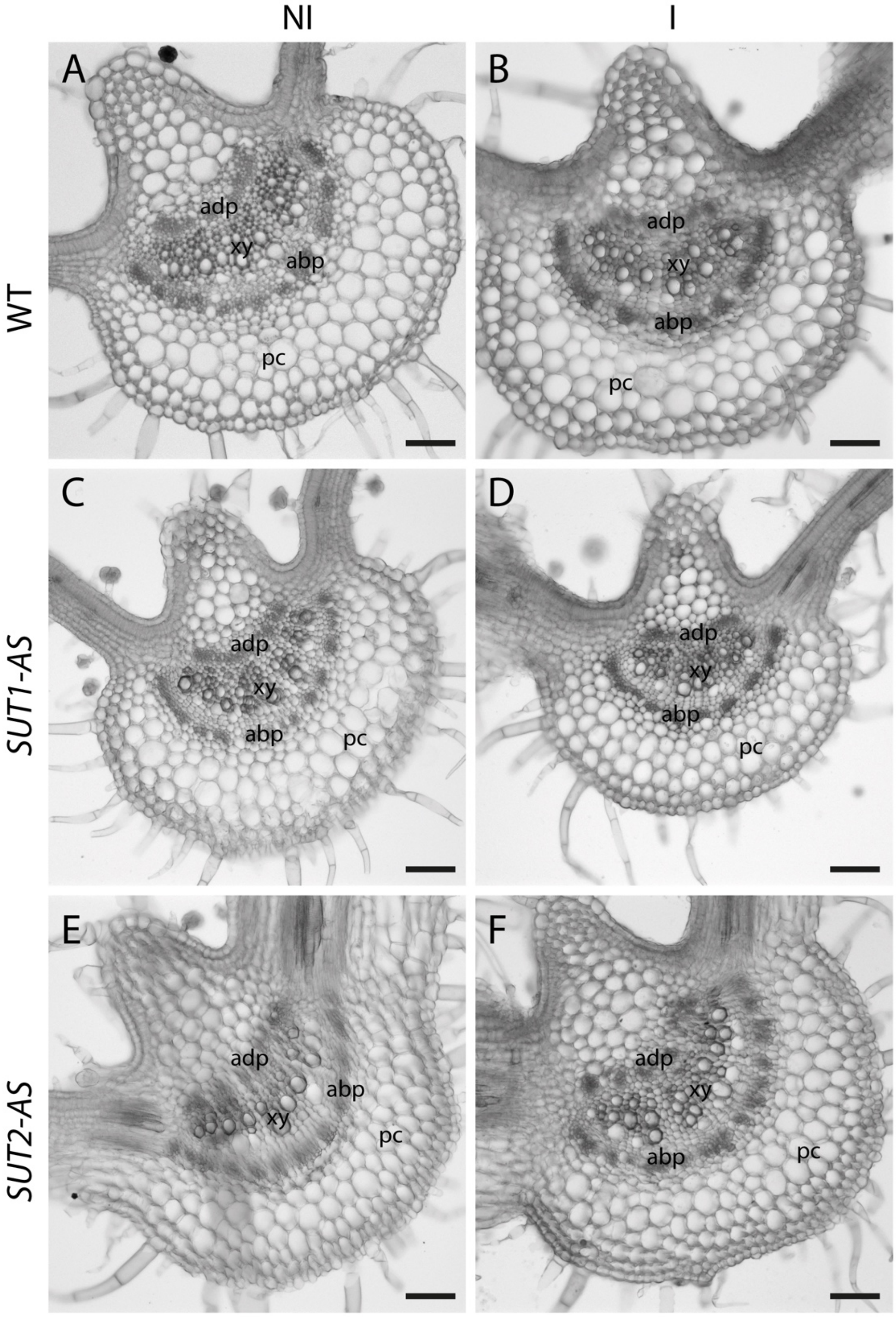

Figure S1. Transversal sections of L1 leaves from not-infected and infected plants. Images were obtained on fresh transverse thin sections of the main vein of L1 leaflet. A,B: Wild-type (WT), C,D: *SUT1*-AS, E,F: *SUT2*-AS transversal main vein sections of not-infected (NI) and infected (IF) plants respectively. Vascular tissues exhibit a typical organization with xylem vessels surrounded by abaxial and adaxial phloem. adp, adaxial phloem, abp, abaxial phloem, xy, xylem, pc, parenchyma cells. Bar, 100 µm.

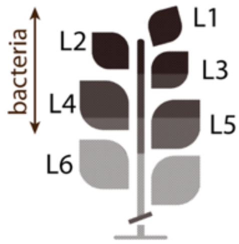

		WT		SUT1-AS		SUT2-AS		ANOVA		
		NI	I	NI	I	NI	I	Inf	G	GxInf
Starch	L1	0.06	0.15	0.07	0.13	0.06	0.23	***	ns	ns
	L4	0.07	0.08	0.05	0.07	0.05	0.05	ns	ns	ns
	L6	0.15	0.07	0.14	0.10	0.10	0.07	ns	ns	ns
Glucose	L1	0.08	0.10	0.08	0.06	0.06	0.08	ns	ns	ns
	L4	0.12	0.14	0.13	0.12	0.10	0.13	ns	ns	ns
	L6	0.10	0.11	0.15	0.16	0.09	0.09	ns	*	ns
Fructose	L1	0.10	0.12	0.11	0.08	0.08	0.09	ns	ns	ns
	L4	0.14	0.15	0.11	0.15	0.13	0.12	ns	ns	ns
	L6	0.11	0.13	0.19	0.22	0.14	0.14	ns	*	ns
Sucrose	L1	0.03	0.05	0.03	0.03	0.02	0.04	ns	ns	ns
	L4	0.04	0.03	0.02	0.02	0.02	0.02	ns	ns	ns
	L6	0.02	0.02	0.03	0.03	0.02	0.03	ns	ns	ns
Sucrose-to-Starch	L1	0.68	0.30	0.44	0.18	0.33	0.15	*	ns	ns
	L4	0.82	0.37	0.57	0.32	0.58	0.50	ns	ns	ns
	L6	0.26	0.27	0.19	0.37	0.19	0.57	ns	ns	ns
Hexoses-to-Sucrose	L1	12.08	8.78	6.61	6.84	10.09	9.05	ns	ns	ns
	L4	5.98	17.37	12.49	16.82	15.88	13.28	ns	ns	ns
	L6	20.60	17.80	14.55	12.01	24.39	8.01	ns	ns	ns

Min [scale] Max

Figure S2. Amounts of sugars and starch in the leaves in response to the phytoplasma infection. Heat map and ANOVA for starch and soluble sugar contents at three leaf levels (L1, L4 and L6), sampled at 18 days after grafting. Left panel: the mean values for each compound and each genotype in not-infected (NI) and infected (I) plants. Data are expressed in nmol mg^{-1} of fresh weight. Right panel: *p* values obtained by two-way ANOVA for each leaf level (*, $P < 0.05$; ***, $P < 0.001$; ns, not significant), with Inf: infection effect, G: genotype effect and G x Inf: genotype per infection interaction effect.

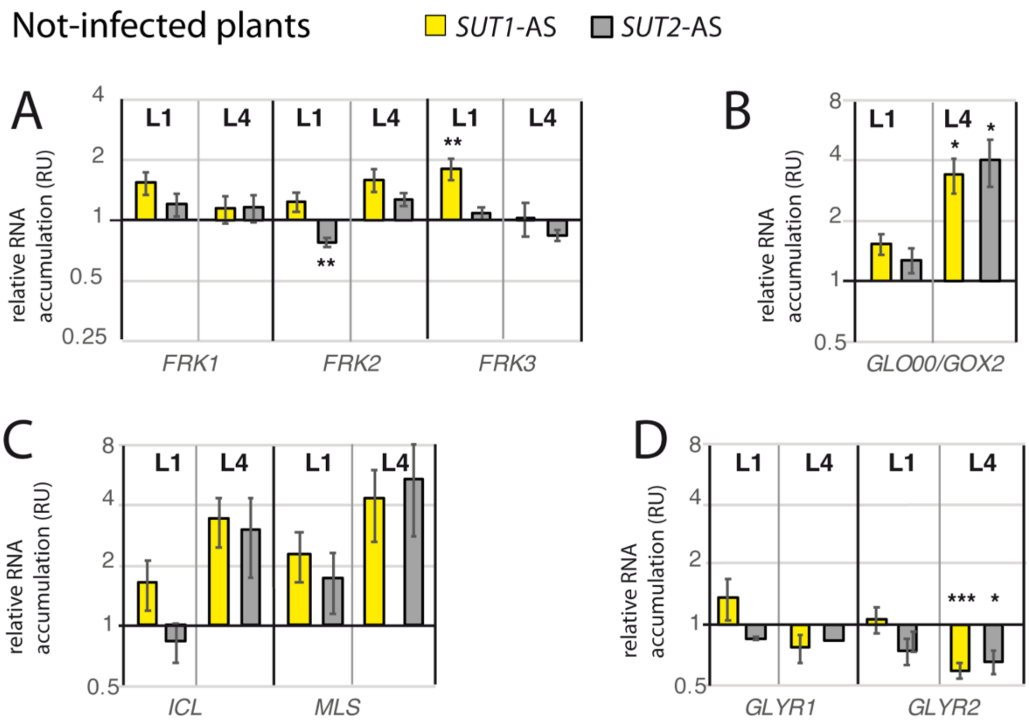

Figure S3. Transcript profiles of genes in not-infected *AS* plants compared to wild-type (WT) plants in L1 and L4 leaves. In A: *FRK* genes, in B, *GLO00/GOX2* gene (marker of the photorespiration), in C, *ICL* and *MLS* genes (markers of the glyoxylate cycle), and in D: *GLYR1* and *GLYR1* genes. Histograms show for each gene mean response +/- *SE* (*n*=4). Y-axis: Relative transcript accumulation, reported to the mean value of WT plants set to 1, Y-axis is drawn with a log_2 scale. RU: relative units for content. *P*-values: *, *P* < 0.05; **, *P* < 0.01; ***, *P* < 0.001. ns, not significant.

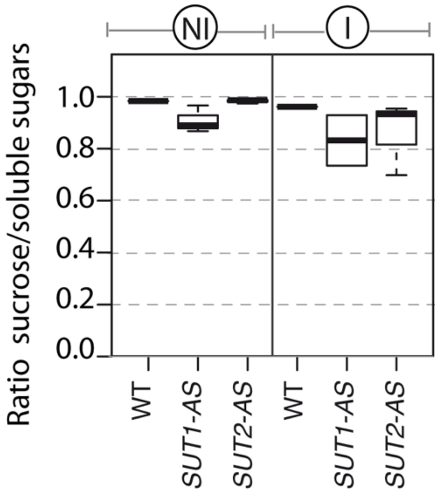

Figure S4. Enrichment of the exudates in phloem sap. The ratio of sucrose relative to soluble sugars (sucrose, fructose and glucose) is 0.97 in non-infected plants (NI) and to 0.95 in infected plants (I), showing that sucrose is the main sugar found in the exudate. High values for sucrose demonstrated that the exudates are enriched in phloem sap exudates [1]. The presence of hexoses could indicate minor contamination by apoplasmic fluids or invertase activity in the exudates during the period of exudation.

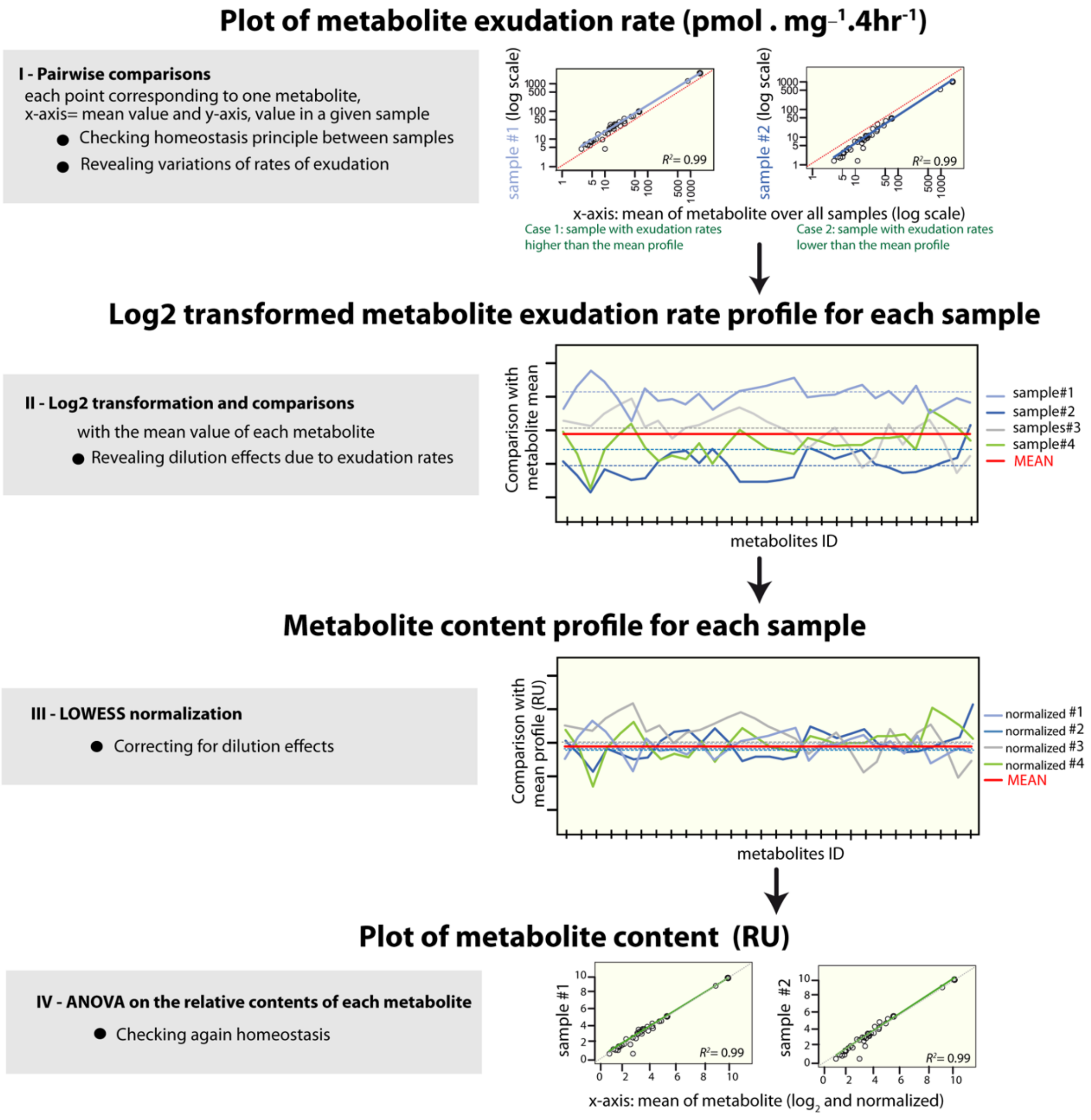

Figure S5. Workflow of metabolite data treatments. Workflow consists in data pairwise comparisons to verify homeostasis between samples, log_2 transformation, LOWESS normalization and statistical analysis. This procedure reveals the variations of exudation rates and corrects for dilution effects. RU: relative units

Supplementary Tables

Table S1. Symptoms after infection by '*Ca* Phytoplasma solani' in WT, *SUT1*-AS and *SUT2*-AS plants Number of plants for each class of symptoms. The classes of symptoms, from 0 to 4, correspond to no symptoms (class 0) to small crook-shaped and chlorotic leaves (class 4) (see details in the main text). Data were obtained on 4 plants. The first visible symptoms appeared at 17 DAG and the symptoms were recorded at 3 dates after grafting (DAG: days after grafting) from 18 to 27 DAG. Above panel: number of plants for each class of symptoms. Bottom panel: Kruskal Wallis tests followed by Dunn test performed on the symptoms level (classes from 0 to 4) for each date.

	**WT**	***SUT1-AS***	***SUT2-AS***	**WT**	***SUT1-AS***	***SUT2-AS***	**WT**	***SUT1-AS***	***SUT2-AS***
Class	**18 DAG**			**24 DAG**			**27 DAG**		
0	0	3	0	0	0	0	0	0	0
1	0	1	0	0	0	0	0	0	0
2	1	0	0	0	2	0	0	1	0
3	1	0	4	1	2	2	0	2	1
3.5	0	0	0	0	0	0	1	1	0
4	2	0	0	3	0	2	3	0	3

**Kruskal Wallis test *p*-value**	**18 DAG**	**24 DAG**	**27 DAG**
	0.0207 *	0.0505	0.0455 *
**Post hoc Dunn test adjusted with the Holm method**	**18 DAG**	**24 DAG**	**27 DAG**
WT vs *SUT1*-AS	0.0304 *	0.0592	0.069
WT vs *SUT2*-AS	0.7188	0.5963	0.7915
SUT1-AS vs *SUT2*-AS	0.054	0.1433	0.0891

Table S2. Exudation rate of metabolites identified in the phloem-enriched exudates of non-infected and infected plants.

I: infected plants; NI: non-infected plants

Rate of exudation Mean +/- *SE*

Mean +/- Standard error for each group (pmol per mg FW per hour of exudation)

		Exudation rate (pmole per mg FW and per hour of exudation)											
		WT NI		WT I		SUT1-AS NI		SUT1-AS I		SUT2-AS NI		SUT2-AS I	
		Mean	SE	Mean	SE	Mean	SE	Mean	SE	Mean	SE	Mean	SE
Amino acids	Alpha-Alanine	2.321	0.458	2.321	0.339	2.321	0.444	2.321	0.321	2.321	1.135	2.321	0.371
	Arginine	0.580	0.139	0.950	0.198	0.286	nd	nd	nd	nd	nd	1.227	0.626
	Asparagine	3.441	0.775	2.406	0.729	3.452	0.743	2.146	0.205	3.466	1.259	4.560	1.642
	Aspartate	2.408	0.786	2.276	0.286	3.289	0.794	2.706	0.502	3.495	0.765	2.652	0.561
	Beta-Alanine	2.916	0.389	1.891	0.184	2.300	0.459	2.174	0.285	1.890	0.291	1.765	0.241
	GABA	6.933	1.308	4.054	0.613	3.736	0.622	4.347	0.874	7.010	1.304	5.730	0.894
	Glutamate	3.504	0.920	2.301	0.214	2.744	0.347	2.943	0.445	4.089	0.697	3.022	0.540
	Glutamine	5.769	1.392	4.610	1.108	4.729	0.908	2.886	0.424	8.106	2.008	7.264	2.881
	Glycine	2.907	0.887	2.670	0.591	2.567	0.492	2.472	0.470	4.475	1.466	2.300	0.599
	Isoleucine	0.887	0.156	1.617	0.367	0.986	0.274	1.779	0.244	1.922	0.248	1.488	0.395
	Leucine	0.989	0.136	1.524	0.314	1.691	0.959	1.544	0.067	2.342	0.443	1.168	0.280
	Lysine	0.755	0.111	0.769	0.145	1.009	0.401	0.673	nd	1.558	0.421	1.030	0.211
	Methionine	0.572	0.113	nd	nd	nd	nd	0.431	0.069	nd	nd	nd	nd
	Methylcysteine	nd	nd	1.091	0.123	nd	nd	1.265	0.153	0.885	0.155	0.819	0.030
	Ornithine	1.110	0.495	1.384	0.372	1.443	0.399	1.676	0.202	1.689	0.717	1.240	0.582
	Phenylalanine	1.092	0.108	1.504	0.504	1.065	0.255	1.391	0.528	1.917	0.680	0.619	0.084
	Proline	4.054	1.025	3.261	0.477	6.537	1.711	3.290	0.882	3.295	0.744	3.722	0.890
	Serine	6.612	1.494	3.488	0.770	2.714	0.409	3.515	0.465	8.047	3.115	4.471	0.809
	Threonine	1.920	0.433	1.363	0.183	1.117	0.203	1.496	0.380	2.313	0.583	1.266	0.214
	Tryptophan	nd	nd	nd	nd	nd	nd	1.012	0.257	nd	nd	0.699	0.101
	Tyrosine	1.477	0.186	1.209	0.107	1.330	0.265	1.257	0.271	1.477	0.374	1.094	0.344
	Valine	1.355	0.276	1.194	0.463	1.922	0.460	0.955	0.128	2.193	0.453	1.982	0.656
Organic acids	Citrate	8.025	1.366	4.213	0.846	5.127	0.620	4.567	1.149	5.755	1.136	6.005	1.134
	Fumarate	5.898	0.325	nd	nd	6.338	nd	29.776	nd	10.542	5.608	5.484	nd
	Glycerate	3.676	0.112	nd	nd	6.287	nd	17.295	nd	5.632	0.890	6.855	1.607
	Glycolate	17.365	6.311	31.327	7.813	37.659	5.558	28.811	5.783	21.399	2.817	22.144	3.672
	Glyoxylate	22.706	7.412	40.195	12.461	27.128	6.274	63.957	13.482	14.291	2.510	68.462	10.907
	Malate	10.636	1.191	7.855	1.462	9.522	1.557	7.592	1.358	7.864	1.184	10.042	2.432
	Quinate	4.138	0.431	2.755	0.466	2.841	0.589	3.251	0.304	2.477	0.489	6.169	1.252
	Succinate	8.458	1.372	5.209	1.193	8.042	0.926	7.783	2.651	7.710	1.705	10.675	0.039
Sugars	Fructose	3.927	nd	2.064	nd	2.051	0.319	2.912	1.246	1.223	0.097	7.767	0.951
	Galactinol	0.613	0.303	nd	nd	0.949	0.464	1.118	nd	0.540	nd	0.976	nd
	Glucose	4.886	1.724	5.813	1.325	5.168	1.065	7.815	2.550	2.459	0.444	15.983	3.880
	MyoInositol	15.900	1.855	13.699	3.656	15.941	2.301	12.824	2.786	9.390	1.075	13.860	1.945
	Sucrose	412.814	87.787	132.365	29.061	78.492	14.360	95.539	26.568	221.936	48.907	277.699	71.641
	Xylose	16.275	5.886	20.397	2.767	22.195	4.346	11.203	6.105	13.329	1.256	17.226	4.128

Student's *t*-test (*p*-value)

p-value color scale: 0.001 | 0.005 | 0.01 | 0.05 | 1

	INFECTION EFFECT per genotype			Genotype effect in NI		Genotype effect in I plants	
	WT I vs WT NI	SUT1-AS I vs NI	SUT2-AS I vs NI	SUT1-AS vs WT NI	SUT2-AS vs WT NI	SUT1-AS vs WT I	SUT2-AS vs WT I
Alpha-Alanine	0.499	0.211	0.216	0.462	0.163	0.525	0.638
Arginine	0.207	nd	nd	nd	nd	nd	0.714
Asparagine	0.350	0.108	0.611	0.994	0.986	0.800	0.212
Aspartate	0.870	0.546	0.391	0.446	0.341	0.455	0.545
Beta-Alanine	0.027	0.808	0.744	0.330	0.056	0.419	0.684
GABA	0.058	0.580	0.434	0.048	0.967	0.784	0.138
Glutamate	0.198	0.735	0.242	0.454	0.622	0.215	0.235
Glutamine	0.521	0.083	0.819	0.583	0.358	0.223	0.404
Glycine	0.828	0.896	0.200	0.771	0.364	0.795	0.670
Isoleucine	0.068	0.083	0.359	0.746	0.004	0.722	0.817
Leucine	0.100	0.893	0.085	0.195	0.010	0.968	0.430
Lysine	0.953	nd	0.521	0.458	0.073	nd	0.415
Methionine	nd	nd	nd	nd	nd	nd	nd
Methylcysteine	nd	nd	0.812	nd	nd	0.426	0.187
Ornithine	0.680	0.709	0.681	0.612	0.534	0.593	0.834
Phenylalanine	0.268	0.711	0.075	0.911	0.098	0.902	0.096
Proline	0.476	0.094	0.718	0.224	0.560	0.977	0.633
Serine	0.088	0.225	0.288	0.039	0.685	0.978	0.396
Threonine	0.445	0.382	0.178	0.173	0.599	0.742	0.769
Tryptophan	nd	nd	nd	nd	nd	nd	nd
Tyrosine	0.290	0.862	0.624	0.649	0.999	0.851	0.671
Valine	0.789	0.171	0.791	0.288	0.131	0.577	0.485
	0.101	0.658	0.187				
Citrate	0.030	0.652	0.883	0.077	0.225	0.826	0.225
Fumarate	nd	nd	nd	nd	0.495	nd	nd
Glycerate	nd	nd	0.502	nd	0.217	nd	nd
Glycolate	0.196	0.293	0.877	0.033	0.570	0.799	0.305
Glyoxylate	0.385	0.047	0.001	0.683	0.209	0.235	0.114
Malate	0.172	0.365	0.456	0.580	0.125	0.897	0.454
Quinate	0.052	0.641	0.014	0.129	0.027	0.586	0.016
Succinate	0.099	0.907	0.405	0.806	0.738	0.356	0.052
Fructose	nd	0.459	0.002	nd	nd	nd	nd
Galactinol	nd	nd	nd	0.636	nd	nd	nd
Glucose	0.684	0.334	0.009	0.889	0.105	0.465	0.059
MyoInositol	0.616	0.412	0.067	0.989	0.010	0.852	0.971
Sucrose	0.007	0.598	0.544	0.003	0.082	0.366	0.081
Xylose	0.481	0.258	0.254	0.436	0.537	0.181	0.557

Ratio

color scale: MIN | 1.000 | MAX

(lower value in blue, higher value in red)

	INFECTION EFFECT per genotype			Genotype effect in NI		Genotype effect in I plants	
	WT I vs WT NI	SUT1-AS I vs NI	SUT2-AS I vs NI	SUT1-AS vs WT NI	SUT2-AS vs WT NI	SUT1-AS vs WT I	SUT2-AS vs WT I
Alpha-Alanine	1.303	1.786	0.565	0.616	2.008	0.844	0.871
Arginine	1.637			0.493			1.292
Asparagine	0.699	0.622	1.316	1.003	1.007	0.892	1.895
Aspartate	0.945	0.823	0.759	1.366	1.451	1.189	1.165
Beta-Alanine	0.649	0.945	0.934	0.789	0.648	1.149	0.933
GABA	0.585	1.163	0.817	0.539	1.011	1.072	1.414
Glutamate	0.657	1.073	0.739	0.783	1.167	1.279	1.313
Glutamine	0.799	0.610	0.896	0.820	1.405	0.626	1.576
Glycine	0.918	0.963	0.514	0.883	1.539	0.926	0.861
Isoleucine	1.823	1.804	0.774	1.112	2.167	1.100	0.920
Leucine	1.541	0.913	0.499	1.709	2.368	1.013	0.767
Lysine	1.018	0.667	0.661	1.336	2.063	0.875	1.339
Methionine							
Methylcysteine			0.926			1.159	0.750
Ornithine	1.246	1.161	0.734	1.300	1.522	1.211	0.896
Phenylalanine	1.378	1.306	0.323	0.976	1.757	0.925	0.412
Proline	0.804	0.503	1.129	1.612	0.813	1.009	1.141
Serine	0.528	1.295	0.556	0.410	1.217	1.008	1.282
Threonine	0.710	1.339	0.547	0.582	1.204	1.098	0.929
Tryptophan							
Tyrosine	0.819	0.945	0.740	0.901	1.000	1.039	0.905
Valine	0.882	0.497	0.904	1.419	1.619	0.800	1.660
Citrate	0.525	0.891	1.043	0.639	0.717	1.084	1.425
Fumarate		4.698	0.520	1.075	1.787		
Glycerate		2.751	1.217	1.710	1.532		
Glycolate	1.804	0.765	1.035	2.169	1.232	0.920	0.707
Glyoxylate	1.770	2.358	4.791	1.195	0.629	1.591	1.703
Malate	0.739	0.797	1.277	0.895	0.739	0.967	1.278
Quinate	0.666	1.144	2.491	0.687	0.598	1.180	2.239
Succinate	0.616	0.968	1.385	0.951	0.912	1.494	2.049
Fructose	0.526	1.420	6.349	0.522	0.312	1.411	3.763
Galactinol		1.179	1.808	1.547	0.880		
Glucose	1.190	1.512	6.500	1.058	0.503	1.344	2.750
MyoInositol	0.862	0.804	1.476	1.003	0.591	0.936	1.012
Sucrose	0.321	1.217	1.251	0.190	0.538	0.722	2.098
Xylose	1.253	0.505	1.292	1.364	0.819	0.549	0.845

Fold change (log2)

color scale: MIN | 0.000 | MAX

(negative values in blue, positive values in red)

	INFECTION EFFECT per genotype			Genotype effect in NI		Genotype effect in I plants	
	WT I vs WT NI	SUT1-AS I vs NI	SUT2-AS I vs NI	SUT1-AS vs WT NI	SUT2-AS vs WT NI	SUT1-AS vs WT I	SUT2-AS vs WT I
Alpha-Alanine	0.382	0.837	-0.824	-0.699	1.006	-0.244	-0.200
Arginine	0.711			-1.019			0.370
Asparagine	-0.516	-0.686	0.396	0.004	0.010	-0.165	0.922
Aspartate	-0.082	-0.282	-0.399	0.450	0.537	0.250	0.221
Beta-Alanine	-0.625	-0.082	-0.099	-0.342	-0.626	0.201	-0.099
GABA	-0.774	0.218	-0.291	-0.892	0.016	0.101	0.499
Glutamate	-0.607	0.101	-0.437	-0.353	0.223	0.355	0.393
Glutamine	-0.324	-0.712	-0.158	-0.287	0.491	-0.676	0.656
Glycine	-0.123	-0.054	-0.960	-0.180	0.622	-0.111	-0.215
Isoleucine	0.866	0.851	-0.370	0.153	1.116	0.138	-0.120
Leucine	0.624	-0.131	-1.003	0.774	1.244	0.019	-0.383
Lysine	0.026	-0.584	-0.597	0.418	1.045	-0.192	0.421
Methionine							
Methylcysteine			-0.111			0.213	-0.414
Ornithine	0.318	0.216	-0.446	0.378	0.606	0.276	-0.158
Phenylalanine	0.462	0.385	-1.631	-0.035	0.813	-0.112	-1.280
Proline	-0.314	-0.991	0.175	0.689	-0.299	0.013	0.191
Serine	-0.923	0.373	-0.848	-1.285	0.283	0.011	0.358
Threonine	-0.495	0.421	-0.869	-0.782	0.268	0.134	-0.106
Tryptophan							
Tyrosine	-0.289	-0.082	-0.434	-0.151	0.000	0.056	-0.144
Valine	-0.182	-1.008	-0.146	0.504	0.695	-0.322	0.731
Citrate	-0.930	-0.167	0.061	-0.646	-0.480	0.116	0.511
Fumarate		2.232	-0.943	0.104	0.838		
Glycerate		1.460	0.283	0.774	0.616		
Glycolate	0.851	-0.386	0.049	1.117	0.301	-0.121	-0.501
Glyoxylate	0.824	1.237	2.260	0.257	-0.668	0.670	0.768
Malate	-0.437	-0.327	0.353	-0.160	-0.436	-0.049	0.354
Quinate	-0.587	0.194	1.317	-0.542	-0.741	0.238	1.163
Succinate	-0.699	-0.047	0.469	-0.073	-0.134	0.579	1.035
Fructose	-0.928	0.506	2.667	-0.937	-1.683	0.497	1.912
Galactinol		0.237	0.854	0.630	-0.184		
Glucose	0.251	0.597	2.701	0.081	-0.991	0.427	1.459
MyoInositol	-0.215	-0.314	0.562	0.004	-0.760	-0.095	0.017
Sucrose	-1.641	0.284	0.323	-2.395	-0.895	-0.470	1.069
Xylose	0.326	-0.986	0.370	0.448	-0.288	-0.864	-0.244

Table S3. Content of metabolites in the phloem-enriched exudates of non-infected and infected plants (log_2 transformed and normalized values).

Content : mean +/- SE

Mean +/- SE for each group (in relative units, RU)

Metabolite content (expressed in relatve units)

		WT C		WT I		LeSUT1 -AS C		LeSUT1 -AS I		LeSUT2 -AS C		LeSUT2 -AS I	
		Mean	SE	Mean	SE	Mean	SE	Mean	SE	Mean	SE	Mean	SE
Amino acids	AlphaAlani	2.4944	0.2197	3.0038	0.1741	2.0609	0.5953	2.5282	0.2911	2.7257	0.2406	2.9590	0.2105
	Asparagine	3.4445	0.2633	3.1618	0.3078	3.4557	0.2624	2.9587	0.2921	3.2636	0.3708	3.4723	0.3554
	Aspartate	2.7180	0.2568	3.3349	0.1437	3.3874	0.1263	3.3208	0.1013	3.4958	0.1720	3.1504	0.1362
	BetaAlanin	3.5052	0.1591	3.0668	0.1853	3.0860	0.2849	3.0325	0.1302	2.7140	0.2104	2.7987	0.1611
	GABA	4.3962	0.2268	4.2762	0.1241	3.9259	0.1005	4.1159	0.1713	4.6466	0.0999	4.0383	0.0914
	Glutamate	3.4308	0.2008	3.4276	0.1633	3.2689	0.1785	3.6527	0.1587	3.7474	0.2269	3.4891	0.1806
	Glutamine	3.8238	0.2908	4.2272	0.2220	4.1693	0.2025	3.5868	0.1174	4.6777	0.2294	3.9097	0.3290
	Glycine	3.1610	0.1548	3.2545	0.3295	3.3040	0.2401	3.2339	0.2178	3.7032	0.1553	3.1388	0.3185
	Isoleucine	1.9107	0.0981	2.4601	0.1734	2.1763	0.0661	2.5626	0.1967	2.4778	0.2521	2.5375	0.2332
	Leucine	2.0972	0.1036	2.3241	0.1741	2.6149	0.2935	2.3096	0.5168	2.6578	0.1424	2.2311	0.0830
	Lysine	1.8198	0.1160	1.7912	0.1050	1.8217	0.3536	1.4568	nd	2.0222	0.1411	1.6529	0.0395
	Ornithine	1.7579	0.4141	2.2549	0.1989	2.1504	0.2110	2.5834	0.3250	1.8954	0.2655	2.2349	0.4269
	Phenylalan	2.3467	0.1389	1.9434	0.3308	2.1202	0.2386	1.8471	0.1912	2.1293	0.3283	1.4870	0.1306
	Proline	3.3681	0.3381	3.8425	0.1439	4.3158	0.2347	3.6358	0.2444	3.4243	0.2441	3.3615	0.1923
	Serine	4.2591	0.1173	3.7541	0.3039	3.4836	0.0480	3.8812	0.2971	4.2768	0.3507	3.7694	0.2224
	Threonine	2.9023	0.1199	2.2516	0.3531	2.0467	0.1970	2.5227	0.0979	2.7445	0.0799	2.4620	0.0979
	Tyrosine	2.7189	0.2705	2.0965	0.1111	2.4976	0.1406	1.8523	0.2475	2.0729	0.1570	2.1252	0.1725
	Valine	2.4457	0.0659	2.1240	0.3709	2.7747	0.0675	2.0710	0.1325	2.5863	0.1505	2.6389	0.3227
Organic acic	Citrate	4.6061	0.1810	4.2207	0.1313	4.4410	0.0802	4.2571	0.1787	4.3822	0.0495	3.9650	0.1785
	Glycolate	5.7058	0.3411	6.5876	0.1356	6.8741	0.0878	6.4219	0.1029	6.8158	0.1409	6.0690	0.1308
	Glyoxylate	6.0923	0.4705	6.8162	0.1722	6.4579	0.1135	7.5741	0.1244	6.1955	0.0940	7.5002	0.0817
	Malate	5.1053	0.1670	5.0686	0.1307	5.0802	0.1243	5.0810	0.1308	5.0139	0.1290	4.7415	0.1988
	Quinate	3.8423	0.3190	3.7049	0.0988	3.6011	0.2635	3.7858	0.0015	3.0139	0.1613	3.8154	0.2638
	Succinate	4.6399	0.1564	4.6024	0.2361	5.0001	0.1420	4.7114	0.2618	4.8840	0.2172	4.3326	0.2692
Sugars	Galactinol	1.8303	0.4307	nd	nd	1.5996	0.4171	1.7907	nd	1.4440	nd	2.0537	nd
	MyoInosito	5.8695	0.2080	5.5371	0.1521	5.6174	0.1565	5.4069	0.1622	5.4787	0.1404	5.3519	0.1763
	Sucrose	9.0952	0.0557	8.9412	0.0228	8.9867	0.0203	8.8248	0.0261	9.0444	0.0275	8.8725	0.0147
	Xylose	5.8213	0.3059	6.2151	0.1643	5.9935	0.1774	5.3013	0.2293	6.1048	0.0576	5.7042	0.5026

Student's *t*-test (*p*-value)

p-value color scale: 0.001 0.005 0.01 0.05

		INFECTION EFFECT per genotype			Genotype effect in NI plants		Genotype effect in I plants	
		WT I vs WT NI	LeSUT1-AS I vs LeSUT1-AS NI	LeSUT2-AS I vs LeSUT2-AS NI	LeSUT1-AS NI vs WT NI	LeSUT2-AS NI vs WT NI	*LeSUT1* -AS I vs WT I	*LeSUT2* -AS I vs WT I
Amino acids	AlphaAlani	0.1493	0.4533	0.4930	0.4087	0.5190	0.2323	0.8835
	Asparagine	0.4986	0.2790	0.6952	0.9804	0.6900	0.6741	0.5258
	Aspartate	0.0492	0.6883	0.1414	0.0375	0.0271	0.9393	0.3723
	BetaAlanin	0.1006	0.8496	0.7511	0.1973	0.0111	0.8816	0.2932
	GABA	0.6385	0.3577	0.0007	0.0823	0.3323	0.4543	0.1565
	Glutamate	0.9901	0.1308	0.3840	0.5580	0.3167	0.3396	0.8043
	Glutamine	0.2835	0.0288	0.0856	0.3932	0.0398	0.0397	0.4370
	Glycine	0.8017	0.8383	0.1423	0.6106	0.0318	0.9583	0.8072
	Isoleucine	0.0143	0.1975	0.8681	0.1355	0.0480	0.7059	0.7966
	Leucine	0.2594	0.6587	0.0546	0.0672	0.0078	0.9735	0.6466
	Lysine	0.9053	nd	0.2028	0.9951	0.2870	nd	0.3428
	Ornithine	0.3386	0.2863	0.4921	0.4181	0.7787	0.3917	0.9648
	Phenylalan	0.2093	0.4377	0.0971	0.4121	0.4814	0.7966	0.2094
	Proline	0.1989	0.0748	0.8476	0.0480	0.8951	0.4781	0.0631
	Serine	0.1471	0.2159	0.2452	0.0001	0.9627	0.7724	0.9683
	Threonine	0.0509	0.2131	0.0482	0.0028	0.2947	0.5982	0.4940
	Tyrosine	0.0949	0.0479	0.8737	0.5355	0.0612	0.3361	0.8953
	Valine	0.1605	0.0018	0.8722	0.0068	0.3859	0.8808	0.3901
Organic acic	Citrate	0.1028	0.2998	0.0256	0.4205	0.2557	0.8840	0.2649
	Glycolate	0.0253	0.0059	0.0019	0.0061	0.0109	0.3467	0.0155
	Glyoxylate	0.1118	0.0000	0.0000	0.3310	0.7685	0.0032	0.0031
	Malate	0.8634	0.9966	0.2863	0.9059	0.6724	0.9474	0.1909
	Quinate	0.7087	0.6249	0.0461	0.6602	0.0504	0.6694	0.6320
	Succinate	0.8970	0.3690	0.2522	0.1138	0.3796	0.8258	0.5898
Sugars	Galactinol	nd	nd	nd	0.7373	nd	nd	nd
	MyoInosito	0.2121	0.3710	0.5840	0.3519	0.1454	0.5675	0.4379
	Sucrose	0.0186	0.0004	0.0001	0.0917	0.4285	0.0047	0.0241
	Xylose	0.2389	0.0975	0.2376	0.6110	0.2580	0.0331	0.2256

Fold change (log2)

Fold change color scale: MIN 0.000 MAX
(negative values in blue, positive values in red)

		INFECTION EFFECT per genotype			Genotype effect in NI plants		Genotype effect in I plants	
		WT I vs WT NI	LeSUT1-AS I vs LeSUT1-AS NI	LeSUT2-AS I vs LeSUT2-AS NI	LeSUT1-AS NI vs WT NI	LeSUT2-AS NI vs WT NI	*LeSUT1* -AS I vs WT I	*LeSUT2* -AS I vs WT I
Amino acids	AlphaAlani	0.5094	0.4673	0.2332	-0.4335	0.2313	-0.4757	-0.0449
	Asparagine	-0.2826	-0.4970	0.2087	0.0113	-0.1809	-0.2031	0.3104
	Aspartate	0.6169	-0.0666	-0.3454	0.6694	0.7778	-0.0140	-0.1845
	BetaAlanin	-0.4384	-0.0535	0.0846	-0.4192	-0.7912	-0.0343	-0.2682
	GABA	-0.1200	0.1900	-0.6083	-0.4703	0.2504	-0.1602	-0.2378
	Glutamate	-0.0033	0.3838	-0.2583	-0.1619	0.3166	0.2251	0.0615
	Glutamine	0.4034	-0.5825	-0.7680	0.3455	0.8539	-0.6405	-0.3175
	Glycine	0.0935	-0.0701	-0.5644	0.1430	0.5422	-0.0206	-0.1156
	Isoleucine	0.5494	0.3864	0.0597	0.2656	0.5672	0.1026	0.0774
	Leucine	0.2270	-0.3052	-0.4268	0.5177	0.5607	-0.0145	-0.0931
	Lysine	-0.0286	-0.3650	-0.3693	0.0019	0.2024	-0.3345	-0.1383
	Ornithine	0.4970	0.4329	0.3394	0.3925	0.1375	0.3285	-0.0200
	Phenylalan	-0.4034	-0.2731	-0.6423	-0.2265	-0.2174	-0.0963	-0.4563
	Proline	0.4744	-0.6801	-0.0627	0.9477	0.0562	-0.2067	-0.4809
	Serine	-0.5050	0.3975	-0.5074	-0.7755	0.0177	0.1271	0.0153
	Threonine	-0.6508	0.4760	-0.2825	-0.8557	-0.1579	0.2712	0.2104
	Tyrosine	-0.6224	-0.6454	0.0523	-0.2213	-0.6461	-0.2442	0.0287
	Valine	-0.3217	-0.7037	0.0526	0.3290	0.1406	-0.0530	0.5149
Organic acic	Citrate	-0.3855	-0.1839	-0.4172	-0.1651	-0.2239	0.0365	-0.2557
	Glycolate	0.8818	-0.4523	-0.7468	1.1683	1.1100	-0.1657	-0.5185
	Glyoxylate	0.7239	1.1162	1.3047	0.3656	0.1032	0.7579	0.6840
	Malate	-0.0367	0.0008	-0.2723	-0.0251	-0.0914	0.0124	-0.3270
	Quinate	-0.1374	0.1847	0.8015	-0.2412	-0.8284	0.0809	0.1105
	Succinate	-0.0374	-0.2887	-0.5514	0.3602	0.2441	0.1090	-0.2699
Sugars	Galactinol	nd	0.1911	0.6097	-0.2307	-0.3863	nd	nd
	MyoInosito	-0.3323	-0.2104	-0.1268	-0.2521	-0.3908	-0.1302	-0.1853
	Sucrose	-0.1541	-0.1619	-0.1719	-0.1086	-0.0509	-0.1164	-0.0686
	Xylose	0.3937	-0.6922	-0.4006	0.1722	0.2835	-0.9138	-0.5108

Table S4. List and characteristics of candidate genes.

* Gene pattern of expression, unless specifically indicated, with: l: leaf, yl: young leaf, sl: senescent leaf, s: stem, r: roots, s: seeds, yf: young fruit, mf: mature fruits, f: fruit, fl: flowers. TCMV: tomato chlorotic mottle virus infection. *P.s.*: *Pseudomonas syringae* pv. tomato DC3000, *F.s.*: *Fusarium lycopersici. R. s.: Ralstonia solanacearum. Ca.* P. s.: *'Candidatus* Phytoplasma solani', *RKN:* root-knot nematode. CC: companion cells. SE: sieve elements.

Gene name	Arabidopsis ortholog	Function	Expression*	References
Defence related genes				
PR1a/P4	At4g33720 (putative *PR1*)	Pathogenesis related protein	l; SA dependent defence gene, upregulation by *Ca.* P. s. infection	[2]–[4]
PR2a	At4g16260 (β–1,3-glucanase)	Pathogenesis related protein (β-1,3-glucanase)	l; SA dependent defence gene, upregulation by *Ca.* P. s. infection	[2], [3]
CAS2	At2g31960 (GLS03)	Putative callose synthase	l, No response to *Ca.* P. s. infection	De Marco, personal communication
CAS7	At1g06490 (*CAS7*)	Putative callose synthase	l; Upregulation by *Ca.* P. s. infection	[5]
Vascular marker				
PP2	At4g19840 (*PP2-A1*)	Phloem protein 2 (lectin)	l, phloem; Downregulation by *Ca.* P. s. infection	[5]
Sugar metabolism				
FRK1	At5g51830 (*FRK1*)	Cytosolic fructokinase	l, st, r, f, vascular tissues	[6]–[8]
FRK2	At1g06030 (*FRK2/6*)	Cytosolic fructokinase	s, r, f, vascular tissues Increased protein amount in response to *R.s.*	[7], [9], [10]
FRK3	At1g66430 (*FRK3/6*)	Plastidial fructokinase	r, l, st, s. vascular tissues. Increased protein amount in response to TCMV infection	[7], [11]–[13]
SUSY1	At5g20830 (*SUS1*)	Sucrose synthase	st, vascular tissues (xylem)	[14]
SUSY3	At3g43190 (*SUS4*)	Sucrose synthase	r	[14]
Sugar transport				
SUT1	At1g22710 (*SUC2*)	Sucrose transporter	l, st, source organs mRNA in CC, protein in SE	[15]–[17]
SUT2	At2g02860 (*SUC3*)	Sucrose transporter	l, st, sink organs mRNA in CC, Protein in SE. Upregulation by *RKN*	[17], [18]
SWEET2a	At3g14770 (*SWEET2*)	Sugar facilitator	l, st, r, fl, Upregulation by *RKN*	[19], [20]
SWEET5b	At5g62850 (*SWEET5*)	Sugar facilitator	l, fl, Upregulation in leaves and roots by *RKN*	[19], [20]
SWEET10c	At5g50790 (*SWEET10*)	Sugar facilitator	l, fl, Upregulation by sugars, salt and temperature	[19]
SWEE11a	At3g48740 (*SWEET11*)	Sugar facilitator	l, fl, Upregulation by sugars, salt and temperature	[19]

SWEE12a	At5g23660 (*SWEET12*) At3g48740 (*SWEET11*)	Sugar facilitator	l, fl, Upregulation by sugars, salt and temperature	[19]
Glyoxylate cycle and photorespiration				
ICL	At3g21720 (*ICL*)	Isocitrate lyase	yl, sl. Protein in yl. and sl., less in mat. leaves	[21], [22]
MLS	At5g03860 (*MLS*)	Malate synthase	yl, sl. Protein in yl. and sl., lower activity in the roots by *R.s.* infection	[21]–[23]
GLO40/GOX1/ SlGlo1/ TomGlo.1	At3g14420 (*GOX1*) At3g14415 (*GOX2*)	Glycolate oxidase	l, Decreased activity in response to *F. l.*. No response to *P.s.*	[4], [24], [25]
GLO00/GOX2 /SlGlo2	At3g14420 (*GOX1*) At3g14415 (*GOX2*)	Glycolate oxidase	yf, l, Decreased activity in response to *F.s.* Upregulation by *P.s* . Involved in the photorespiration	[4], [24], [25]
GLO50/GOX3/ SlGlo3	At4g18360 (*GOX3*)	Glycolate oxidase	L, Decreased activity in response to *F. l.*	[24]
GLYR1	At3g25530 (*GLYR1*)	Glyoxylate reductase	L, Increased activity by *F. l.* infection	[24]
GLYR2	At1g17650 (*GLYR2*)	Glyoxylate reductase	L, Increased activity by *F. l.* infection	[24]

Table S5. Two-way ANOVA of the expression of candidate genes in not-infected and infected plants.

The ANOVA was determined on gene expression data obtained at 3 leaf levels for the 3 genotypes (WT, *SUT1*-AS, *SUT2*-AS). G: Genotype; L: leaf position; GxL= Genotype per leaf position interaction. *p*-value (2-way ANOVA): *, *P* <0.05, **; *P* <0.01; ***, *P* <0.001. *n*=72.

Gene	G	L	GxL	G	L	GxL
	**Not-infected**			**Infected**		
SUS1	0.0132 *	4.07e-05 ***	0.4750	0.2085	3.83e-05 ***	0.6739
SUS3	0.2073	0.0053 **	0.7242	0.0057 **	0.0006 ***	0.0888
FRK1	0.1837	0.0004 ***	0.6185	0.0004 ***	8.59e-05 ***	0.6609
FRK2	0.0017 **	0.0085 **	0.2882	0.3586	0.0001 ***	0.5379
FRK3	0.0226 *	0.0031 **	0.3118	0.1911	0.0033 **	0.5541
CAS2	0.0055 **	2.16e-07 ***	0.0239 *	0.0124 *	2.63e-06 ***	0.3743
CAS7	0.0747	2.32e-10 ***	0.9559	0.5643	4.46e-06 ***	0.6072
PR1a	0.0584	0.0854	0.5372	0.0122 *	0.2525	0.3696
PR2a	0.4755	0.0265 *	0.4345	0.1518	0.2261	0.5719
SWEET2a	0.0089 **	0.0008 ***	0.5491	0.0268*	0.0016**	0.5703
SWEET5b	0.1581	6.03e-06 ***	0.7338	0.3010	9.37e-06 ***	0.0906
SWEET10c	0.2010	0.0007 ***	0.5131	0.4201	5.20e-05 ***	0.8110
SWEET11a	0.4628	0.0033 **	0.4946	0.0417 *	3.57e-05 ***	0.7356
SWEET12a	0.3217	0.0004 ***	0.5877	0.0497 *	0.0001***	0.8447
PP2	0.5485	3.74e-07 ***	0.5769	0.472	2.79e-06 ***	0.6045
ICL	0.0640	0.0370 *	0.6605	0.0145 *	0.3298	0.5561
MLS	0.0568	0.4368	0.9470	0.0002 ***	0.9635	0.9771
GLO00/GOX2	0.0035 **	0.3770	0.6526	0.0142 *	0.0025 **	0.4767
GLO40/GOX1	0.5239	0.0206 *	0.9951	0.3176	0.0009 ***	0.7862
GLO50/GOX3	0.2170	0.2787	0.9831	0.5420	0.1109	0.5444
GLYR1	0.4658	0.0318 *	0.4585	0.6818	0.0355 *	0.6063
GLYR2	0.0012 **	0.0025 **	0.0951	0.0356 *	0.6020	0.0756

Table S6. Correlations in gene expression.

The *Pearson* correlation was determined on the full gene expression dataset obtained at the three leaf levels (L1, L4 and L6) of the infected and non-infected plants of the 3 genotypes (WT, *SUT1*-AS and *SUT2*-AS). *n*=72.

**Correlations**	***Pearson* correlation**	**Pairwise two-sided *p*-values**	**Adjusted *p*-values**
Glycolysis and peroxisome metabolism			
FRK1 - ICL	-0.406	0.000	0.019
FRK1 - GLYR2	-0.384	0.001	0.037
FRK3 - GLO40/GOX1	0.394	0.009	0.028
FRK3 - GLO50/GOX3	0.416	0.005	0.014
FRK3 - GLYR1	0.401	0.030	0.022
SUS1 - GLO40/GOX1	0.438	<0.0001	0.006
SUS3 - GLO40/GOX1	0.450	<0.0001	0.004
SUS3 - GLO50/GOX3	0.470	<0.0001	0.002
SUS3 - GLYR1	0.473	<0.0001	0.001
Glycolysis			
SUS1 - FRK1	0.446	<0.0001	0.004
SUS1 - FRK2	0.649	<0.0001	<0.0001
SUS1 - FRK3	0.687	<0.0001	<0.0001
SUS1 - SUS3	0.518	<0.0001	0.000
SUS3 - FRK2	0.486	<0.0001	0.001
SUS3 - FRK3	0.599	<0.0001	<0.0001
FRK1 - FRK2	0.527	0.000	0.000
FRK2 - FRK3	0.724	0.000	0.000
Peroxisomal metabolism			
GLO00/GOX2 - ICL	0.644	<0.0001	<0.0001
GLO00/GOX2 - MLS	0.835	<0.0001	<0.0001
ICL- MLS	0.734	<0.0001	<0.0001
GLO50/GOX3- GLO40/GOX1	0.626	<0.0001	<0.0001
GLO50/GOX3- GLYR1	0.653	<0.0001	<0.0001
GLO40/GOX1- GLYR1	0.884	<0.0001	<0.0001
Others			
CAS7 - PP2	0.906	<0.0001	<0.0001
CAS7- FRK3	0.734	<0.0001	<0.0001
PP2 - FRK3	0.742	<.0001	<.0001

Table S7. List of Primers.

When more than one sequence was identified, primers amplify each variant. E (%): Efficiency of the primers.

Gene	Accession no. (NCBI)	Forward (F) and Reverse (R) primers (5'-3')	E (%)	Reference
**Stolbur markers**				
STOL-rRNA	AF248959	F: AGGGTAGCTAAAGCGTAAGC<br>R: CATCAACCCTACCTTAGACG	92.4	[26]
STOL-gDNA	AM990981	F: ATTTGATGAAACACGCTGGATAA<br>R: TCCCTGGAACAATAAAAGTYGCA	99.2	[27]
**Tomato candidate genes**				
Sugar transport				
SUT1	NM_001302901.2	F: TTCCATAGCTGCTGGTGTTC<br>R: TACCAGAAATGGGTCCACAA	109.5	Forward: [16]
SUT2	NM_001247392.2<br>NM_001313887.1	F: CCTACAGCGTCCCTTTCTCT<br>R: CGATACAACCATCTGAGGTACAA	109.8	Forward: [16]
SWEET2a	XM_004244249.3<br>XM_019215006.1<br>XM_010326104.2	F: TGTTTGCCTCCCCACTGTTA<br>R: TAGGAAGGTGGAGAGGGACA	95.3	This study
SWEET5b	NM_001320885.1	F: TGTACACCTCACCACTGACC<br>R: CATGCCCAAACAATGCCATT	93.8	This study
SWEET10c	XM_004235291.3	F: GATGGCTATTGCTGGTCATTGG R: ACCCTGGCTTTCTTTGGTGC	103.2	[19]
SWEET11a	XM_004235278.3	F: CTACGCACCAAAGAAAGCCAG<br>R: GTCTCACAATGCCTAAGGGAGC	102.4	[19]
SWEET12a	XM_004235286.3	F: TCTCACAATGCCTAAGGGTGC<br>R: ACCAAACAAAGCCAGGGTCC	108.2	[19]
Sugar metabolism				
SUS1	XM_019211274.1	F: CTGCTGAGTGAATGAAGGTC<br>R: GATACTAATGGAAATGAAACAC	103.6	[14]
SUS3	XM_019214635.2	F: GGTTTCTGTCTGATTGTTATCC<br>R: ACAGAAGGGAAAAATGGCAAA	89	[14]
FRK1	NM_001246964.2<br>XM_010319082.1	F: CTCCGTTACATATCTGATCCTT<br>R: GACAGCATTGAAGTCACCTT	105.4	[28]
FRK2	NM_001246959.2	F: TTGTTGGTGCCCTTCTAACCA<br>R: ACGATGTTTCTATGCTCCTCCCT	110.9	[28]
FRK3	NM_001247467.2	F: GTGGTGCATTGACCGTGATG<br>R: GGTCGGATGGTATTATGCAACTG	99.2	[11]
Stress markers				
CAS2	XM_026031249.1	F: CGTCAGTGGGCTTGAAGAAG<br>R: GCACAAAAGACTGAGAGGCA	102.1	This study
CAS7	XM_010325327.2<br>XM_010325328.2	F: GGGACTTGGAATTTGGGATT<br>R: CTCTGATACGAATGGGAACCA	101.9	[5]
PR1a	AJ011520.1	F: CTGTAGGCAACTGGGTTGGA<br>R: TCAATCCGATCCCACTTATCATTT	95.7	This study
PR2a	NM_001247869.2	F: AGGGCTAAACGATGCAGGAT<br>R: GCCAACCACTTTCCGATACA	93.8	This study
Vascular markers				
PP2	XM_004233183.4	F: TGAAGGTGGGAACTGGAAGA<br>R: CTGGGCTTAACGCCAAATC	94.7	[5]

Glyoxylate cycle				
ICL	NM_001246949.2	F: CGACACGTTTGCCAAGGATT<br>R: ACACCATTGCTCCTCTCCTC	101.7	This study
MLS	XM_010320762.1<br>XM_004236297.3<br>XM_004236298.3	F: GTGGCTGATCGGAGGGTG<br>R: GGCCTCTCATTAAATTCTCCCA	94.3	This study
GLO00/GOX2	NM_001347977.1<br>NM_001347978.1	F: ATCAGGCGTGTTTATTGGGC<br>R: CTTCAGTGAACGACAGCCAC	99.6	This study
GLO40/GOX1	NM_001307942.1<br>XM_004243141.3<br>XM_010325318.2	F: CTGTCGCTCACTGAAGGAGA<br>R: GTACATGCTCACAACCTTGGG	98.0	This study
GLO50/GOX3	XM_010326599.2<br>XM_010326600.2<br>XM_010326601.2<br>XM_004244918.3<br>XM_019215374.1	F: CCTGTTTTCCTTGACGGTGG<br>R: CTAACTCCTGCCTCCCCATC	99.2	This study
GLYR1	NM_001246903.2	F: AGCAGAAGGACATGAGGCTG<br>R: AAGTCAAGGTCTCCCAAGCC	98.7	This study
GLYR2	NM_001246907.2	F: GTGCTCCAATGTATGCCGTT<br>R: AACCCAGAGCTAGACGAAGG	97.6	This study
**Tomato reference genes**				
UBI3<br>*Ubiquitin*	NM_001346406.1	F: TCGTAAGGATGCCCTAATGCTGA<br>R: CAATCGCCTCCAGCCTTGTTGTAA	100.2	[29]
UPL3<br>*E3 ubiquitin-protein ligase*	XM_004230989.3<br>XM_010317077.2	F: AGCGCTGATCCCTCATTGCAT<br>R: GCTGTTTACAAATTCCTCCGAGG	98.5	[5]
PGK<br>*Phosphoglycerate kinase*	NM_001329592.1<br>NM_001329591.1	F: TCTACAAGGCCCAAGGTTATG<br>R: GCAGCAAACTTGTCCGCAATC	97.1	[30]
UrK<br>*UMP-CMPkinase 3*	NM_001309811.1	F: TGGTAAGGCACCCAATGTGCTAA<br>R: ATCATCGTCCCATTCTCGGAACCA	99.7	[29]

References

[1] S. Dinant and J. Kehr, “Sampling and analysis of phloem sap,” in *Plant Mineral Nutrients: Methods and Protocols*, vol. 953, F. Maathuis, Ed. Humana Press, USA, 2013, pp. 185–194.

[2] J. A. L. van Kan, M. H. A. J. Joosten, C. A. M. Wagemakers, G. C. M. van den Berg-velthuis, and P. J. G. M. De Wit, “Differential accumulation of mRNAs encoding extracellular and intracellular PR proteins in tomato induced by virulent and avirulent races of *Cladosporium fulvum*,” *Plant Mol. Biol.*, vol. 69247, no. 20, pp. 513–527, 1992.

[3] J. N. Ahmad, J. Renaudin, and S. Eveillard, “Expression of defence genes in Stolbur phytoplasma infected tomatoes, and effect of defence stimulators on disease development,” *Eur. J. Plant Pathol.*, vol. 139, no. 1, pp. 39–51, 2014.

[4] G. J. Ahammed, X. Li, G. Zhang, H. Zhang, J. Shi, C. Pan, J. Yu, and K. Shi, “Tomato photorespiratory glycolate-oxidase-derived H_2O_2 production contributes to basal defence against *Pseudomonas syringae*,” *Plant Cell Environ.*, vol. 41, no. 5, pp. 1126–1138, 2018.

[5] F. De Marco, L. Pagliari, F. Degola, S. V. Buxa, A. Loschi, S. Dinant, R. Le Hir, H. Morin, S. Santi, and R. Musetti, “Combined microscopy and molecular analyses show phloem occlusions and cell wall modifications in tomato leaves in response to ‘*Candidatus* Phytoplasma solani,’” *J. Microsc.*, vol. 263, no. 2, pp. 212–225, 2016.

[6] Y. Kanayama, N. Dai, D. Granot, M. Petreikov, A. Schaffer, and A. B. Bennett, “Divergent fructokinase genes are differentially expressed in tomato,” *Plant Physiol.*, vol. 113, no. 4, pp. 1379–1384, 1997.

[7] H. Damari-Weissler, M. Kandel-Kfir, D. Gidoni, A. Mett, E. Belausov, and D. Granot, “Evidence for intracellular spatial separation of hexokinases and fructokinases in tomato plants,” *Planta*, vol. 224, no. 6, pp. 1495–1502, 2006.

[8] O. Stein, F. Secchi, M. A. German, H. Damari-Weissler, R. Aloni, N. M. Holbrook, M. A. Zwieniecky, and D. Granot, “The tomato cytosolic fructokinase FRK1 is important for phloem fiber development,” *Biol. Plant.*, vol. 62, no. 2, pp. 353–361, 2018.

[9] H. Damari-Weissler, S. Rachamilevitch, R. Aloni, M. A. German, S. Cohen, M. A. Zwieniecki, N. M. Holbrook, and D. Granot, “LeFRK2 is required for phloem and xylem differentiation and the transport of both sugar and water,” *Planta*, vol. 230, no. 4, pp. 795–805, 2009.

[10] D. Dahal, D. Heintz, A. Van Dorsselaer, H. P. Braun, and K. Wydra, “Pathogenesis and stress related, as well as metabolic proteins are regulated in tomato stems infected with *Ralstonia solanacearum*,” *Plant Physiol. Biochem.*, vol. 47, no. 9, pp. 838–846, 2009.

[11] M. A. German, I. Asher, M. Petreikov, N. Dai, A. A. Schaffer, and D. Granot, “Cloning, expression and characterization of *LeFRK3*, the fourth tomato (*Lycopersicon esculentum* Mill.) gene encoding fructokinase,” *Plant Sci.*, vol. 166, no. 2, pp. 285–291, 2004.

[12] O. Stein, H. Damari-Weissler, F. Secchi, S. Rachamilevitch, M. A. German, Y. Yeselson, R. Amir, A. Schaffer, N. M. Holbrook, R. Aloni, M. A. Zwieniecki, and D. Granot, “The tomato plastidic fructokinase SlFRK3 plays a role in xylem development,” *New Phytol.*, vol. 209, no. 4, pp. 1484–1495, 2016.

[13] L. S. T. Carmo, A. M. Murad, R. O. Resende, L. S. Boiteux, S. G. Ribeiro, J. V. Jorrín-Novo, and A. Mehta, “Plant responses to tomato chlorotic mottle virus: Proteomic view of the resistance mechanisms to a bipartite begomovirus in tomato,” *J. Proteomics*, vol. 151, pp. 284–292, 2017.

[14] S. Goren, S. C. Huber, and D. Granot, “Comparison of a novel tomato sucrose synthase, SlSUS4, with previously described SlSUS isoforms reveals distinct sequence features and differential expression patterns in association with stem maturation,” *Planta*, vol. 233, no. 5, pp. 1011–1023, 2011.

[15] C. Kuhn, W. P. Quick, A. Schulz, J. W. Riesmeier, U. Sonnewald, and W. B. Frommer, "Companion cell-specific inhibition of the potato sucrose transporter SUT1," *Plant, Cell Environ.*, vol. 19, no. 10, pp. 1115–1123, 1996.

[16] A. Hackel, N. Schauer, F. Carrari, A. R. Fernie, B. Grimm, and C. Kühn, "Sucrose transporter LeSUT1 and LeSUT2 inhibition affects tomato fruit development in different ways," *Plant J.*, vol. 45, no. 2, pp. 180–192, 2006.

[17] D. Zhao, Y. You, H. Fan, X. Zhu, Y. Wang, Y. Duan, Y. Xuan, and L. Chen, "The role of sugar transporter genes during early infection by root-knot nematodes," *Int. J. Mol. Sci.*, vol. 19, no. 1, 2018.

[18] L. Barker, C. Kühn, A. Weise, A. Schulz, C. Gebhardt, B. Hirner, H. Hellmann, W. Schulze, J. M. Ward, and W. B. Frommer, "SUT2, a putative sucrose sensor in sieve elements," *Plant Cell*, vol. 12, no. July, pp. 1153–1164, 2000.

[19] C.-Y. Feng, J.-X. Han, X.-X. Han, and J. Jiang, "Genome-wide identification, phylogeny, and expression analysis of the *SWEET* gene family in tomato," *Gene*, vol. 573, no. 2, pp. 261–272, 2015.

[20] D. Zhao, Y. You, H. Fan, X. Zhu, Y. Wang, Y. Duan, Y. Xuan, and L. Chen, "The role of sugar transporter genes during early infection by root-knot nematodes," *Int. J. Mol. Sci.*, vol. 19, no. 1, p. 302, 2018.

[21] B. Nieri, A. Ciurli, L. Pistelli, S. M. Smith, and A. Alpi, "Glyoxylate cycle enzymes in seedlings and in mature plants of tomato (*Lycopersicon esculentum* Mill.)," *Plant Sci.*, vol. 129, pp. 39–47, 1997.

[22] F. Famiani, A. Paoletti, A. Battistelli, S. Moscatello, Z. Chen, R. C. Leegood, and R. P. Walker, "Phosphoenolpyruvate carboxykinase, pyruvate orthophosphate dikinase and isocitrate lyase in both tomato fruits and leaves, and in the flesh of peach and some other fruits," *J. Plant Physiol.*, vol. 202, pp. 34–44, 2016.

[23] X. Y. Fan, W. P. Lin, R. Liu, N. H. Jiang, and K. Z. Cai, "Physiological response and phenolic metabolism in tomato (Solanum lycopersicum) mediated by silicon under Ralstonia solanacearum infection," *J. Integr. Agric.*, vol. 17, no. 10, pp. 2160–2171, 2018.

[24] B. D. Sanwal and E. R. Waygood, "Glycolic acid oxidase and fusariose wilt of tomatoes," *Can. J. Bot.*, vol. 41, no. 1, pp. 55–63, 1963.

[25] K. Ohta, K. Kanahama, and Y. Kanayama, "Enhanced expression of a novel dioxygenase during the early developmental stage of tomato fruit," *J. Plant Physiol.*, vol. 162, pp. 697–702, 2005.

[26] S. Santi, S. Grisan, A. Pierasco, F. De Marco, and R. Musetti, "Laser microdissection of grapevine leaf phloem infected by Stolbur reveals site-specific gene responses associated to sucrose transport and metabolism," *Plant. Cell Environ.*, vol. 36, no. 2, pp. 343–355, 2013.

[27] C. Pelletier, P. Salar, J. Gillet, G. Cloquemin, P. Very, X. Foissac, and S. Malembic-Mahler, "Triplex real-time PCR assay for sensitive and simultaneous detection of grapevine phytoplasmas of the 16SrV and 16SrXII-A groups with an endogenous analytical control," *Vitis*, vol. 48, no. 2, pp. 87–95, 2009.

[28] M. A. German, N. Dai, I. Chmelnitsky, I. Sobolev, Y. Salts, R. Barg, A. A. Schaffer, and D. Granot, "LeFRK4, a novel tomato (*Lycopersicon esculentum* Mill.) fructokinase specifically expressed in stamens," *Plant Sci.*, vol. 163, no. 3, pp. 607–613, 2002.

[29] T. Mascia, E. Santovito, D. Gallitelli, and F. Cillo, "Evaluation of reference genes for quantitative reverse-transcription polymerase chain reaction normalization in infected tomato plants: RT-qPCR normalization in infected tomato," *Mol. Plant Pathol.*, vol. 11, no. 6, pp. 805–816, 2010.

[30] H. Ghareeb, Z. Bozsó, P. G. Ott, and K. Wydra, "Silicon and *Ralstonia solanacearum* modulate expression stability of housekeeping genes in tomato," *Physiol. Mol. Plant Pathol.*, vol. 75, no. 4, pp. 176–179, 2011.
